# *Bacillus subtilis* remains translationally active after CRISPRi-mediated replication initiation arrest

**DOI:** 10.1128/msystems.00221-24

**Published:** 2024-03-28

**Authors:** Vanessa Muñoz-Gutierrez, Fabián A. Cornejo, Katja Schmidt, Christian K. Frese, Manuel Halte, Marc Erhardt, Alexander K. W. Elsholz, Kürşad Turgay, Emmanuelle Charpentier

**Affiliations:** 1 Max Planck Unit for the Science of Pathogens, Berlin, Germany; 2 Institute of Microbiology, Leibniz Universität Hannover, Hannover, Germany; 3 Humboldt-Universität zu Berlin, Institute of Biology – Molecular Microbiology, Berlin, Germany; 4 Institute of Biology, Humboldt-Universität zu Berlin, Berlin, Germany; Universiteit Leiden, Leiden, the Netherlands

**Keywords:** *Bacillus subtilis*, replication, *oriC*, DnaA boxes, CRISPRi, translation

## Abstract

Initiation of bacterial DNA replication takes place at the origin of replication (*oriC*), a region characterized by the presence of multiple DnaA boxes that serve as the binding sites for the master initiator protein DnaA. This process is tightly controlled by modulation of the availability or activity of DnaA and *oriC* during development or stress conditions. Here, we aimed to uncover the physiological and molecular consequences of stopping replication in the model bacterium *Bacillus subtilis*. We successfully arrested replication in *B. subtilis* by employing a clustered regularly interspaced short palindromic repeats interference (CRISPRi) approach to specifically target the key DnaA boxes 6 and 7, preventing DnaA binding to *oriC*. In this way, other functions of DnaA, such as a transcriptional regulator, were not significantly affected. When replication initiation was halted by this specific artificial and early blockage, we observed that non-replicating cells continued translation and cell growth, and the initial replication arrest did not induce global stress conditions such as the SOS response.

**IMPORTANCE:**

Although bacteria constantly replicate under laboratory conditions, natural environments expose them to various stresses such as lack of nutrients, high salinity, and pH changes, which can trigger non-replicating states. These states can enable bacteria to (i) become tolerant to antibiotics (persisters), (ii) remain inactive in specific niches for an extended period (dormancy), and (iii) adjust to hostile environments. Non-replicating states have also been studied because of the possibility of repurposing energy for the production of additional metabolites or proteins. Using clustered regularly interspaced short palindromic repeats interference (CRISPRi) targeting bacterial replication initiation sequences, we were able to successfully control replication initiation in *Bacillus subtilis*. This precise approach makes it possible to study non-replicating phenotypes, contributing to a better understanding of bacterial adaptive strategies.

## INTRODUCTION

DNA replication duplicates the genome of dividing cells, providing a new, identical copy of DNA for each daughter cell. Replication is therefore an important element of cell growth, chromosome segregation, and cell division. In bacteria, replication initiates at a specific point of the circular chromosome, the origin of replication (*oriC*). This region contains several DNA sequences, known as DnaA boxes, that serve as binding sites for the replication initiator protein DnaA, which, as an ATPase of the AAA+ family, can actively unwind an adenosine and thymine (AT)-rich region within the *oriC* and is required for the the subsequent recruitment of the replication machinery to both strands (1–4).

Regulation of DNA replication initiation relies on the precise control of DnaA binding to the *oriC* region. In *Bacillus subtilis*, several mechanisms have evolved to inhibit this interaction and ensure proper synchronization of replication (2). Firstly, the SeqA-like protein YabA competitively interferes with DnaA-*oriC* interactions, preventing premature initiation (5–7). Secondly, the Soj/ParA system, in conjunction with Spo0J/ParB, contributes to the spatial separation of the *oriC* region, making it less accessible to DnaA (8, 9). The regulatory protein SirA also acts as a mediator by promoting DnaA-ATP hydrolysis, converting DnaA-ATP to its inactive DnaA-ADP form, thus impeding its binding to *oriC* (10–12). This multi-faceted approach to controlling DnaA binding guarantees the fidelity of the replication process.

Bacteria can use non-replicating and non-growing states to adapt to stressful conditions, such as antibiotic exposure. For instance, exposure to antibiotics can lead to the formation of persister cells, which exhibit reduced growth and translation rates, rendering them insensitive to many antibiotics (13). Bacteria can also activate stress-induced regulatory pathways, such as the toxin-antitoxin systems or alarmone signaling, to promote non-replicative states (14) and inhibit protein synthesis and bacterial growth, conserving and redirecting resources until conditions improve (15).

In this study, we constructed an inducible non-replicating strain by integrating a clustered regularly interspaced short palindromic repeats interference (CRISPRi) system specifically targeting DnaA boxes 6 and 7, which have previously been reported as the key boxes for promoting DNA unwinding during replication initiation in *B. subtilis* (16, 17). This approach offers distinct advantages compared to previous methodologies using thermosensitive mutants of *dnaA* or *dnaB* to study replication and cell division (18–20). This selective targeting of DnaA-specific binding boxes allows to focus solely on characterizing the halt of replication initiation at the *oriC* without interfering with other global roles of DnaA (21, 22). To comprehensively assess the effects of halting replication, we characterized phenotypic changes such as cell morphology and replication status. In addition, we used time-resolved quantitative proteome profiling to assess the intracellular changes during the initiation of replication inhibition. Our results suggest that cells cease replicating without inducing stress response pathways, but continue with specific cellular processes such as translation and cell growth. Our study provides a better understanding of the fundamental role of replication initiation in *B. subtilis* and its physiological consequences. It also provides a basis for exploring non-replicating traits and bacterial response to replication barriers using dCas9 in future studies.

## RESULTS

### An inducible CRISPRi system to block replication initiation

**Fig 1:**
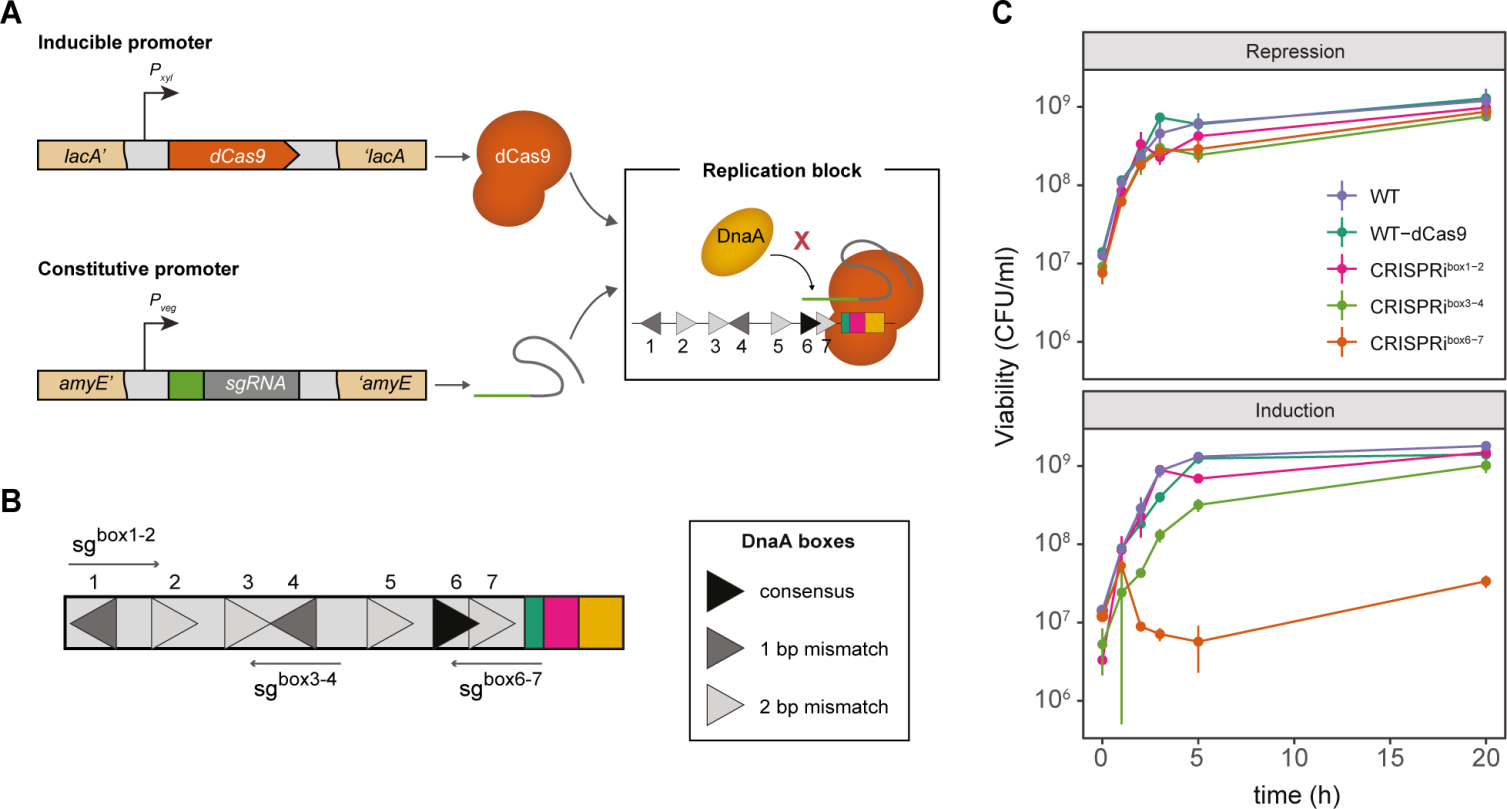
CRISPRi targeting the DnaA boxes 6 and 7 inhibits cell proliferation. (**A**) *B. subtilis* carrying a xylose-inducible dCas9 (orange) is directed to specific DNA targets by constitutively expressed sgRNAs (green) under the control of the *P*_veg_ promoter. The dCas9-sgRNA complex blocks the binding of DnaA (yellow) to the DnaA boxes (triangles). *dcas9* was stably integrated into the *lacA* locus (EC3137), and the sgRNAs were integrated into the *amyE* locus [CRISPRi^box1–2^ (EC3146), CRISPRi^box3–4^ (EC3149), CRISPRi^box6–7^ (EC3147)]. (**B**) DnaA boxes from *B. subtilis* and the selected sgRNA targets. Other elements of the *oriC* are shown: DnaA trios (green), DnaD-binding sites (fuchsia), and the AT-rich region (yellow). (**C**) When induced at the early exponential growth phase, the CRISPRi^box6–7^ strain (EC3147) does not resume growth. Viability of cells (colony forming units [CFU] per milliliter) grown with glucose (dCas9 induction repression) or with xylose (dCas9 induction). The data shown are the mean of three biological replicates; error bars represent standard deviations from the mean.

A previous study characterized the critical role of DnaA boxes 6 and 7 on the *oriC* of *B. subtilis* in orchestrating DNA unwinding and replication initiation (16). We therefore hypothesized that replication initiation could be blocked by inducing dCas9 expression in the presence of single guide RNAs (sgRNAs) specifically designed to target the inactivated nuclease to these boxes and thereby hinder DnaA binding (23) (Fig. 1A). To this end, we constructed *B. subtilis* strains carrying a xylose-inducible dCas9 as well as constitutively expressed sgRNA^box6–7^ designed to target DnaA boxes 6 and 7. In addition, strains encoding sgRNAs designed to target DnaA boxes 1 and 2 or 3 and 4 (sgRNA^box1–2^ and sgRNA^box3–4^, respectively) were constructed as controls (Fig. 1B; Table S1) (16). This system allows conditional expression of dCas9 in the presence of xylose and tight repression when glucose is available.

To assess the impact of this blockade on replication initiation, we examined cell growth during the early logarithmic phase under dCas9 induction or repression conditions. Cells with dCas9 in the absence of sgRNA [wild-type (WT)-dCas9] exhibited normal growth behavior, indicating that dCas9 expression alone does not affect bacterial proliferation (Fig. 1C). Furthermore, cultures repressed by glucose, containing either sgRNA^box1–2^, sgRNA^box3–4^, or sgRNA^box6–7^ showed subtle growth variations compared with the control, while inducing dCas9 with sgRNA^box6-7^ led to a significant decrease in colony forming units (CFU) per milliliter after 3 h compared to the WT (Fig. 1C). Notably, targeting adjacent boxes by inducing the CRISPRi system did not inhibit growth (Fig. 1C). This indicates specific competition between DnaA and dCas9 for binding to DnaA boxes 6 and 7.

The formation of the dCas9-sgRNA^box6–7^ complex could specifically block the formation of DnaA filaments, preventing the opening of the replication bubble associated with initiation. These results confirm the high specificity of DnaA binding to boxes 6 and 7 and demonstrate the potential of the CRISPRi system to modulate critical steps in replication initiation, as previously reported (16).

### The CRISPRi^box6–7^ system inhibits the initiation of replication, leading to replisome disassembly

To study the effect of CRISPRi^box6–7^ blocking replication initiation at the single cell level, we tracked the replisome localization by fusing a translational fluorescent reporter gene with the gene encoding the beta clamp of the polymerase protein, DnaN, at its endogenous genomic locus. DnaN is part of the replisome, making it an excellent reporter for assessing the progression of DNA replication in the cell (5). The green fluorescent protein (GFP)-DnaN fusion protein forms bright foci that assemble and disassemble inside the cells, signaling the start and completion of DNA replication, respectively. Previous studies have shown that no DnaN foci form in the absence of replication, whereas cells undergoing active replication contain between zero and four DnaN foci (5, 24).

**Fig 2:**
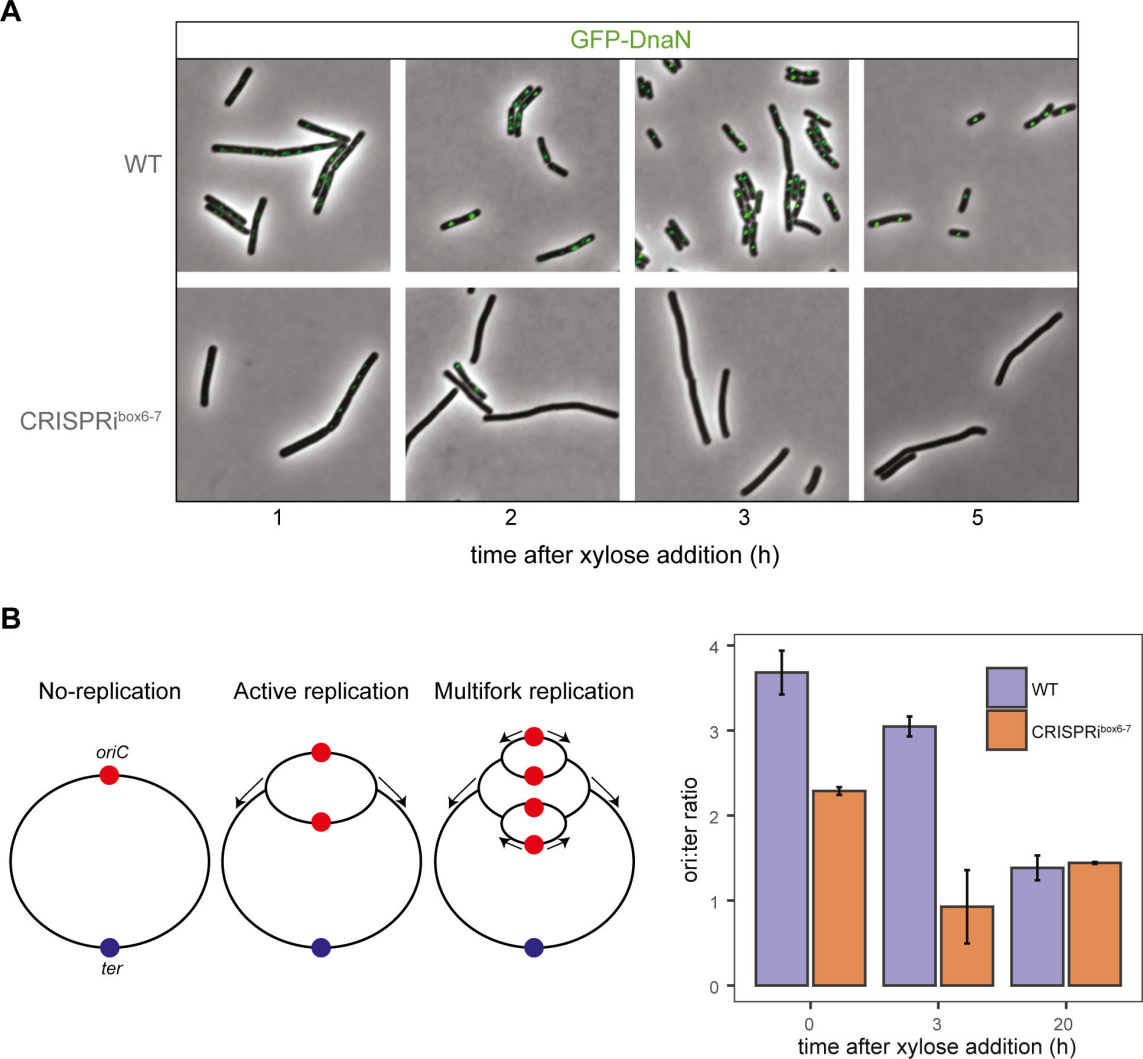
Replication is inhibited in the CRISPRi^box6–7^strain. (**A**) Epifluorescence microscopy of *B. subtilis* cells expressing GFP-DnaN undergoing CRISPRi-mediated replication arrest. WT (EC3237) and CRISPRi^box6–7^ (EC3259) strains were grown in LB (Luria-Bertani) + 1% glucose to an optical density at 600 nm (OD_600_) of 0.1, washed, and resuspended in LB + 1% xylose. Cells were immediately subjected to microscopic analysis at 1, 2, 3, and 5 h after xylose addition. (**B**) Left panel: schematic diagrams of replication states in bacteria. Under nutrient-rich conditions, chromosomes undergo multifork replication and have more than one *oriC* (red) per cell; consequently, their *ori* to *ter* (blue) ratio is higher than under non-replicating conditions. Right panel: *ori:ter* ratios determined by quantitative polymerase chain reaction (qPCR) for WT (EC3017) and CRISPRi^box6–7^ (EC3147) cells. Data in panel **B** represent the means of three independent biological replicates; error bars indicate standard deviations from the mean.

The dynamic localization of GFP-DnaN was assessed under CRISPRi induction (Fig. 2A). The WT strain displayed bright foci, indicating that the replication forks are assembled and cells are actively replicating. Remarkably, in strain CRISPRi^box6–7^, diffusion of foci is already observed after 2 h of xylose induction, suggesting that replication is no longer initiated in these cells (Fig. 2A).

Another method for investigating the status of replication is to analyze the ratio between origin and terminus (*ori:ter* ratio) of the chromosome. Cells that are actively replicating have a ratio greater than 1, while cells containing only one copy of the chromosome, such as non-replicating cells that are in the stationary phase, are expected to have a ratio closer to 1 (Fig. 2B). We determined the *ori:ter* ratios using quantitative polymerase chain reaction (qPCR) (Fig. 2B; Table S3), and observed that blocking the DnaA boxes 6 and 7 resulted in a decrease in DNA replication initiation after 3 h of induction, with an *ori:ter* ratio of 0.88 compared with 2.89 for WT cells. This demonstrates that replication is blocked in the CRISPRi^box6-7^ strain. These observations are consistent with an inhibition of DNA replication initiation and confirm the replisome disassembly observed with the GFP-DnaN reporter in strain CRISPRi^box6–7^.

### Stopping replication initiation results in shape and length defects

**Fig 3:**
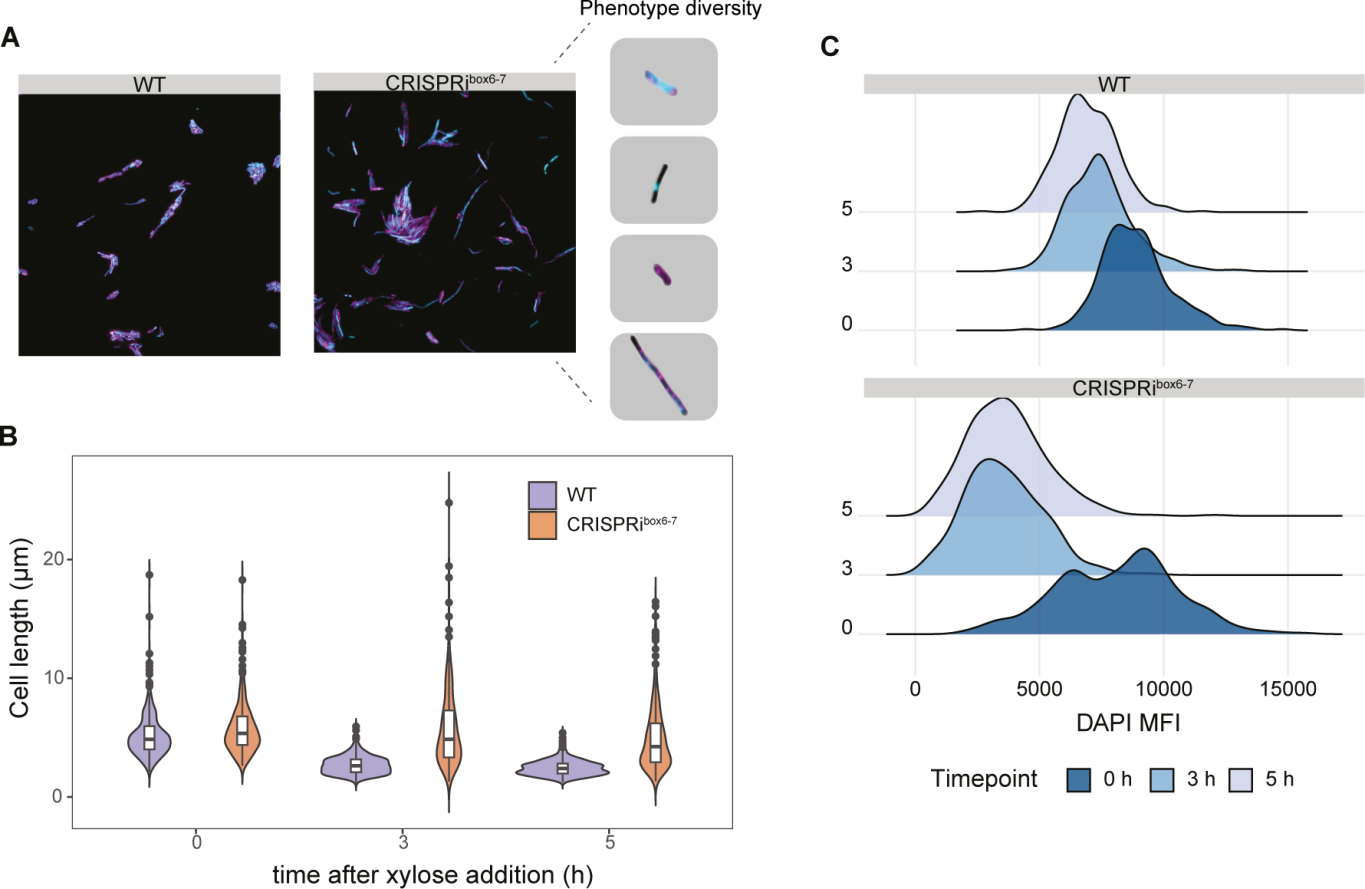
Phenotypic characterization of cells under replication arrest. (**A**) WT (EC3017) and CRISPRi^box6–7^ (EC3147) strains stained with DAPI (cyan) and FM4-64 (magenta) and grown in Luria-Bertani with 1% xylose. Representative picture of a subset of different phenotypes observed by microscopy after 3 h after xylose addition. (**B**) Cell length distribution was quantified from microscopy pictures after 0, 3, and 5 h of xylose induction. *n* = 3, at least 100 cells measured per replicate (**C**) Density plot of the DAPI mean fluorescence intensity (MFI) distribution after 0, 3, and 5 h of xylose induction, as an approximation of the DNA content within the cell in WT and CRISPRi^box6–7^ strains.

We observed that, despite the cessation of cell number increase following CRISPRi induction (Fig. 1C), the optical density (OD) of the culture continued to increase over time, albeit slightly less for strain CRISPRi^box6–7^ (Fig. S1A), implying potential alterations in cell shape or form. We stained the cell membrane and nucleoid with FM4-64 and DAPI (4',6-diamidino-2-phenylindole) to assess morphological changes following replication arrest. Prior to induction, both the WT and CRISPRi^box6–7^ strains exhibited typical exponential phase cell morphology (Fig. S1B). After 5 h in the presence of xylose, WT cells showed reduced length and chaining, consistent with the appearance of WT cells transitioning to the stationary phase (Fig. 3A and B). Conversely, CRISPRi^box6–7^ cells showed increased length compared to the WT cells. Quantifying cell length at 0, 3, and 5 h after xylose induction revealed that non-replicating CRISPRi^box6–7^ cells retain lengths akin to cells in exponential phase, in contrast to WT cells in stationary phase.

In replication-arrested cells, a spectrum of morphological abnormalities was observed, as illustrated in Fig. 3A and B. The irregular cell shapes manifested as anucleate cells, cell bending and elongated forms, reflecting the dynamic consequences of replication disruption. Furthermore, the organization of chromosomal DNA in these cells exhibited marked heterogeneity, in some cases, as single foci or as a diffuse distribution in the cytoplasm.

Notably, a subset of replication-arrested cells displayed reduced DAPI staining, indicating potential variations in chromosomal content. In contrast, WT cells retained a predominantly uniform distribution of DAPI staining. Quantification of mean DAPI fluorescence intensity (MFI) per cell unveiled a distinct bimodal distribution in the CRISPRi strain prior to induction (Fig. 3C). Throughout the experiment, WT cells showed a decrease of DAPI staining, indicative of a cessation of multifork replication upon reaching stationary phase. Conversely, the intensity of DAPI staining decreased considerably in replication-arrested cells after only 3 h.

These results highlight significant alterations in the increase in cell length and decrease in DNA content induced by CRISPRi^box6–7^ replication arrest, resulting in a heterogeneous cell population, including a sub-population of elongated, non-replicating CRISPRi ^box6–7^ cells, distinct from the more homogenous population of WT cells (Fig. 3).

### Inhibition of replication initiation by CRISPRi^box6–7^ does not induce SOS stress response pathways

Bacteria possess the conserved SOS response (reviewed in references 25, 26) to sense and react to DNA stress, which is activated by the accumulation of single-stranded DNA during DNA damage or replication blocks. In *B. subtilis*, the SOS response involves the expression of YneA (27), which is repressed by the transcriptional repressor of the SOS regulon LexA (28). YneA, in turn, hinders cell division during the SOS response by delaying the formation of the FtsZ ring, leading to cell elongation (29).

**Fig 4:**
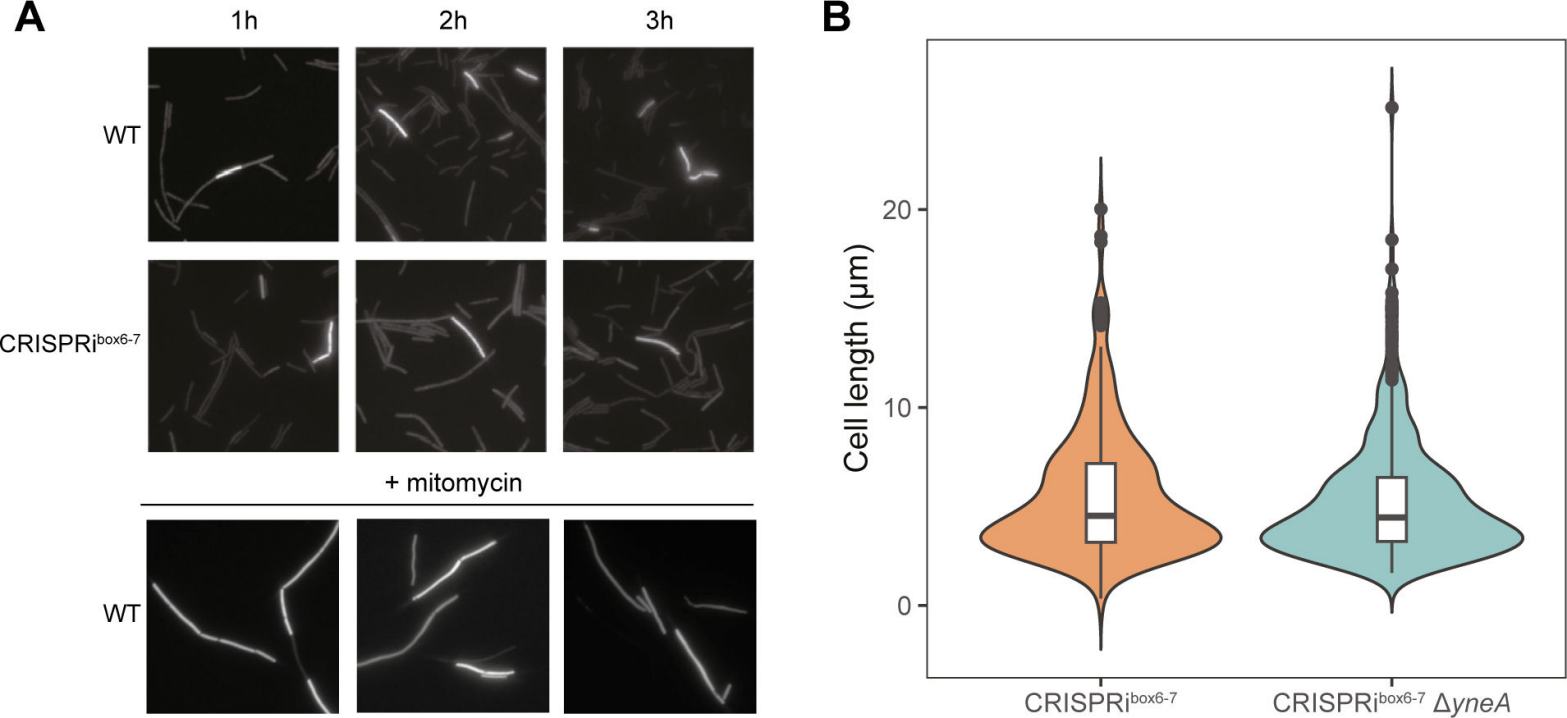
No evidence of SOS response under replication arrest. (**A**) WT (EC3266) and CRISPRi^box6–7^ (EC3272) strains containing ectopic P*_yneA_-gfp* integrations and grown in LB (Luria-Bertani) with 1% xylose. Images are from aliquots taken from cultures constantly growing at 37°C. Cells were subjected to microscopic analysis at 1, 2, and 3 h after xylose addition. Below is a representative picture of cells exposed to mitomycin. (**B**) Quantification of individual cell lengths in CRISPRi^box6–7^ (EC3673) and ∆*yneA* CRISPRi^box6–7^ (EC3698) strains grown in LB with 1% xylose. Strains were grown continuously at 37°C and taken for microscopic analysis 5 h after addition of xylose. Cells show no difference in cell length distribution; the statistical difference was assessed using an unpaired two-sample Wilcoxon test, *P* = 0.28. *n* = 3, ~100 cells per replicate.

To investigate whether the longer cell phenotype results from the activation of the SOS response, we ectopically integrated *gfp* under the control of the *yneA* promoter as a reporter in CRISPRi^box6–7^ and WT strains. GFP fluorescence was monitored at various time points after xylose addition. As a positive control, cells were exposed to mitomycin C, a DNA-damaging agent that induces the SOS response and causes cell elongation (28). Both WT and CRISPRi^box6–7^ strains showed comparable P*_yneA_-gfp* reporter activity after xylose addition (Fig. 4A). This observation indicates that induction of the SOS response is unlikely to be responsible for the increase in cell size upon CRISPRi-mediated replication arrest. Furthermore, we generated strains with a *yneA* deletion and found no impact on the increase in cell size after 5 h of replication arrest (Fig. 4B). Our data indicate that the inhibition of replication initiation can lead to an increase in cell length, independent of the presence of YneA.

### Proteomic characterization of CRISPRi^box6–7^-mediated replication-arrested cells

**Fig 5:**
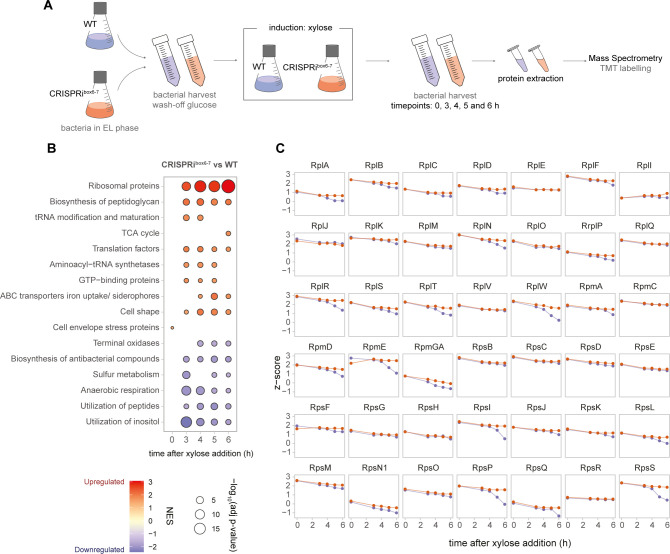
Proteomic characterization shows that translation-related proteins are more abundant in replication-arrested cells after 5 and 6 h of xylose induction. (**A**) An overview of the experimental setup for MS analysis. WT (EC3017) and CRISPRi^box6–7^ (EC3147) cells were harvested at different time points after xylose induction, and their proteins were extracted and quantified by tandem mass tag (TMT) mass spectrometry. EL, early logarithmic phase. (**B**) Gene set enrichment analysis of differentially expressed genes in CRISPRi^box6–7^ and WT strains. Gene categories were obtained from Subtiwiki. The color of dots indicates the normalized enrichment score (NES) values for each process, and the size of the dots represents the −log_10_ of the adjusted *P*-value. (**C**) Profile plots of z-score ribosomal proteins retrieved from Subtiwiki. The purple line indicates the WT strain and the orange line indicates the CRISPRi strain.

To explore the response and physiological state of *B. subtilis* cells upon CRISPRi^box6–7^-mediated replication arrest, we conducted a quantitative mass spectrometry analysis of the proteome, comparing CRISPRi^box6–7^ and WT samples over time (Fig. 5A). We detected 1,784 proteins in all samples, corresponding to a ~42% of *B. subtilis* proteins annotated in UniProt. At time point 0 h, minimal differences were observed in protein abundance profiles between replicating (WT) and non-replicating (CRISPRi^box6–7^) cells (Fig. S2). Here, we would like to highlight a few interesting aspects observed in this experiment (Figs. S3 through S8).

Since the *oriC* is located between the *dnaA* and *dnaN* genes, which are transcribed as a polycistronic mRNA, we investigated whether the binding of dCas9 to the DnaA boxes 6 and 7 affected the expression of these genes. We observed a slight reduction of DnaA levels in CRISPRi^box6-7^ cells compared with WT cells (up to around 54%). However, the levels of DnaN beta clamp, which is required only for replication elongation, were reduced to around 30% compared with WT. This may be explained by the binding of dCas9 to the middle of the polycistronic *dnaA-dnaN* operon (Fig. S3).

Notably, DNA repair and SOS response proteins exhibited no clear distinctions between CRISPRi^box6–7^ and WT cells, with the exception of the differential increase in the nuclease inhibitor DinB upon replication arrest (Fig. S4). These observations collectively suggest the absence of a significant SOS response in CRISPRi^box6–7^-mediated replication-arrested cells. Our analysis revealed no enrichment of SigB-regulated general stress response proteins; the CRISPRi^box6–7^-mediated replication arrest did not affect other related stress systems (Fig. S5).

Given the YneA-independent elongation observed in replication-arrested cells compared to their WT counterparts (Fig. 4), we explored the potential involvement of alternative regulation by DnaA, which could affect cell division. Interestingly, DnaA is also active as a transcription factor, specifically binding to the promoter regions of several cell division genes, including *ftsL* (22). FtsL is an essential but inherently unstable cell division protein (30) whose protein levels rapidly decrease when *ftsL* mRNA levels decline, consequently impeding cell division (26, 31). Although FtsL was not detected in our mass spectrometry analysis, the abundance of RsmH and PbpB, encoded within the same operon, appeared comparable to the WT (Fig. S6). Although this suggests that FtsL levels are not altered during replication arrest, it is difficult to provide conclusive evidence since we were unable to detect this protein.

Other proteins encoded by the DnaA regulon did not change their cellular levels (Fig. S6), suggesting that the DnaA transcription factor activity (22) was not significantly affected by the interference with the DnaA binding to boxes 6 and 7 in the *ori*.

To explore potential reasons for the reduction in cell division, we conducted an analysis of the upregulation or downregulation of cellular processes and pathways, with the aim of providing a comprehensive overview of changes during replication arrest (Fig. 5B). Notably, in CRISPRi^box6–7^ cells compared to WT cells, there was an upregulation of certain cell shape proteins, in particular LytE, as well as a slight upregulation of MreB and MreBH (Fig. S7). Peptidoglycan biosynthesis was also elevated in the CRISPRi^box6–7^ strain compared with the WT strain (Fig. 5B). This could be attributed to a less pronounced downregulation of peptidoglycan precursor biosynthetic enzymes such as MurB, MurC, MurE, and MurG (Fig. S7 and S8).

These observations, together with the absence of significant changes in penicillin-binding proteins (Fig. S7 and S8), suggest a potential disturbance in the balance between peptidoglycan degradation and synthesis, providing a plausible explanation for some of the morphological abnormalities observed (Fig. 3). Furthermore, there were no significant changes in the abundance of proteins involved in cell division and DNA segregation (Fig. S7). Therefore, further research is required to elucidate the mechanism underlying the apparent reduction in cell division in CRISPRi^box6–7^-mediated replication-arrested cells under these growth conditions.

### Protein expression continues in replication-arrested cells

In Fig. 5B, we observed an increased abundance of ribosomal and other translation-related proteins in CRISPRi^box6–7^ strain compared with the WT. Increased translation during replication arrest is a possible mechanism to increase protein and metabolite production and has been studied in *Escherichia coli* (32–34). To assess whether translation-related proteins increase during replication arrest in *B. subtilis*, individual profile plots of these proteins were generated as a function of the z-score (Fig. 5C). Notably, the levels of most ribosomal proteins in the CRISPRi^box6–7^ strain remained relatively constant over time, whereas in WT cells, a subset of the ribosomal proteins decreased mainly at late time points, already entering stationary phase (Fig. S1 and Fig. 5B and C). This might suggest that while WT cells are entering stationary phase, replication-blocked CRISPRi^box6–7^ cells appear to remain in a prolonged exponential phase with continuous, unhinged translation.

To test this hypothesis, we assessed proteomic changes over time in both strains. Both cultures show similar proteome remodeling throughout the experiment (Fig. S8), such as ribosomal protein reduction of different magnitude, as observed in Fig. 5C. Interestingly, some metabolic pathways, such as the TCA cycle, inositol utilization, and sulfur metabolism, show similar trends in both strains, but the magnitude of the upregulation differs. We also observed striking differences, for instance, the lack of downregulation of peptidoglycan biosynthesis in the CRISPRi^box6–7^ strain compared with the WT, which could be related to the differences observed in cell length. These results suggest that the CRISPRi^box6–7^ cells do not pursue a prolonged exponential phase and that they undergo a metabolic rewiring similar to that of WT cells facing the stationary phase.

**Fig 6:**
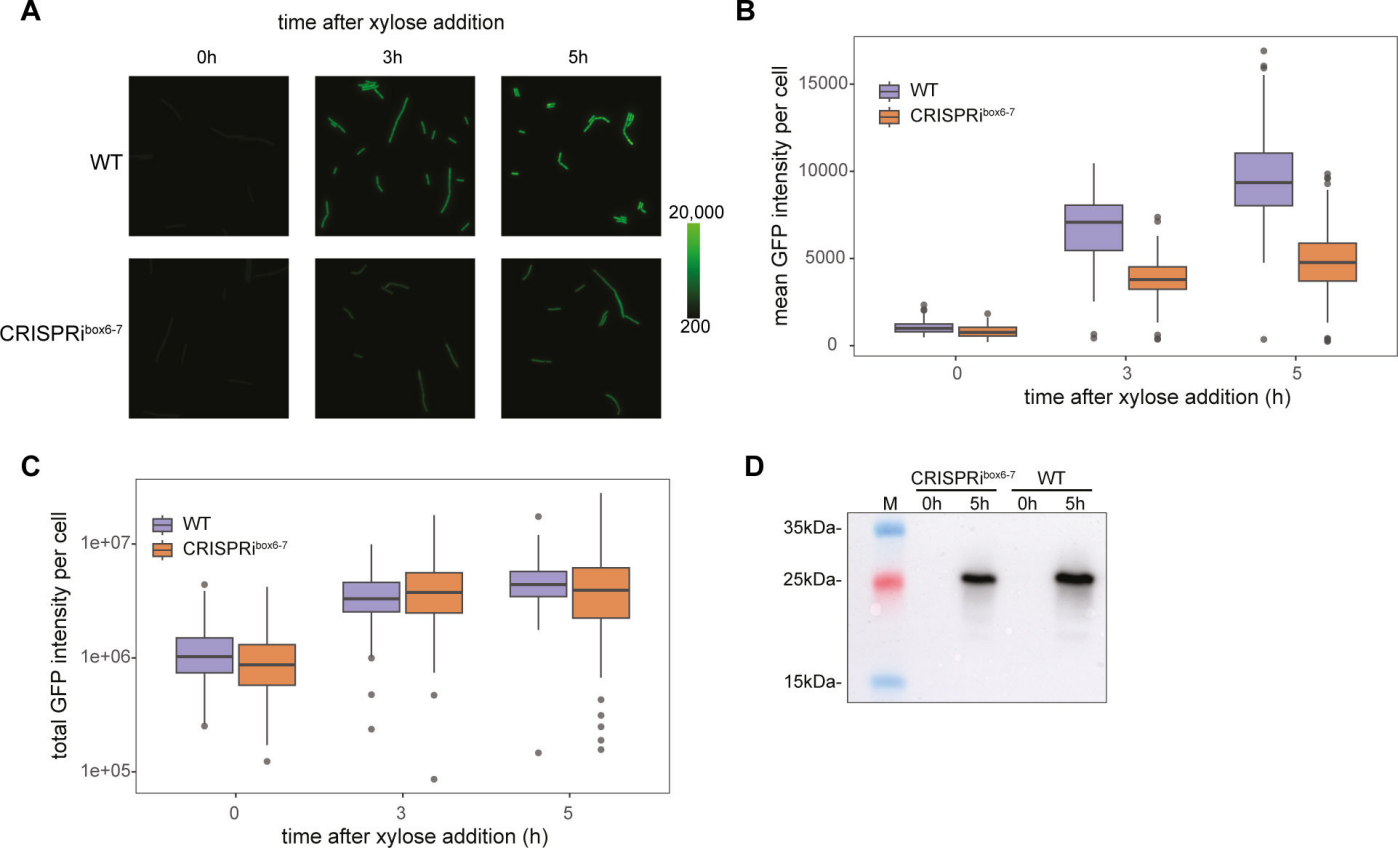
GFP expression continues upon CRISPRi induction. (**A**) WT (EC3700) and CRISPRi^box6–7^ (EC3673) strains containing an ectopic integration of IPTG-inducible *gfp*. Images are from aliquots taken from cultures constantly growing at 37°C in Luria-Bertani at 0, 3, and 5 h after adding 1% xylose and 1 mM IPTG. Representative images of three independent replicates are shown. (**B**) Quantification of mean GFP fluorescence intensity per cell from images of panel A. The WT strain is shown in purple, and the CRISPRi strain is displayed in orange. The boxplots represent the median and interquartile range of three independent biological replicates with at least 100 cells quantified per replicate. (**C**) Microscopic data from (B) have been analyzed to determine the total GFP intensity per cell. The results show that this intensity remains the same in WT and CRISPRi strains in all time points tested of 0, 3, and 5 h after xylose addition. (**D**) Western blot analysis of the two strains confirms that the GFP intensity of the CRISPRi strain after 5 h after xylose addition is similar to the GFP intensity of the WT strain at the same time point. Ten micrograms of total protein extract were transferred onto a polyvinylidene fluoride (PVDF) membrane with a pore size of 0.2 µm. For visualization, we used a horseradish peroxidase (HRP)-linked anti-GFP polyclonal antibody produced in goat.

To further investigate whether the ribosomal protein abundance correlates with increased translation, we ectopically integrated *gfp* under an IPTG (isopropyl β-D-thiogalactopyranoside)-inducible promoter (P*_hyperspank_*) in the WT and CRISPRi strains as a reporter for protein translation. GFP fluorescence was measured by microscopy at 0, 3, and 5 h after co-induction with xylose and IPTG. Here, CRISPRi^box6–7^ blocked cells exhibited lower mean GFP fluorescence intensity than WT cells (Fig. 6A and B). However, when we analyzed the total GFP intensity per cell, taking into account the increased cell length of replication-arrested cells, we observed no difference between the two strains (Fig. 6C). This observation was further supported by western blotting against GFP in bulk cultures after replication arrest (Fig. 6D). Taken together, these results suggest that *B. subtilis* can continue translation after replication initiation is blocked by CRISPRi^box6–7^, achieving levels of exogenous protein production similar to those of the WT strain.

In summary, our results demonstrate the specificity of CRISPRi-mediated replication initiation arrest for DnaA boxes 6 and 7. In addition, this system halts cell proliferation while allowing cellular translation and growth to continue. Notably, these replication-arrested cells do not activate the SOS response or the general stress response.

## DISCUSSION

In this work, we used CRISPRi to selectively block replication initiation in *B. subtilis* by inhibiting the binding of DnaA to specific sequences within the *oriC* without affecting the role of DnaA as a transcriptional regulator (21, 22). DnaA boxes 6 and 7 were the only effective targets of DnaA boxes in the *ori* to inhibit the replication (Fig. 1C), consistent with previous research in *B. subtilis* (16). In contrast to *B. subtilis*, inhibition of any DnaA box within the *oriC* of *E. coli* results in a non-replicating phenotype (35). Based on these observations, we confirm that DnaA boxes 6 and 7 within the *oriC* in *B. subtilis* are essential to trigger replication initiation. It remains to be seen whether this functional hierarchy is a feature common to various bacterial species, given the conserved architecture of *oriC* in *B. subtilis* and its resemblance to other bacteria (30).

The inactivation of the primary origin of replication raises the question of whether a secondary, alternative origin could be activated. To address this possibility, our study included the monitoring of ongoing replication rounds using DnaN-GFP tagging (Fig. 2), indicating that the utilization of an alternative origin is an unlikely event. Moreover, in *B. subtilis*, six DnaA-box clusters have been identified and could potentially serve as alternative origins. However, their role in the initiation of replication is inhibitory, as they sequester DnaA molecules (36). Likewise, a key study by Richardson et al. (16) explored the hierarchical architecture of the *oriC* in *B. subtilis* (16). They integrated *oriN*, a secondary origin of replication, into the chromosome. Activation of *oriN* depended on RepN, conditionally regulated by IPTG (16). Significantly, when the primary *oriC* was non-functional, *B. subtilis* growth relied entirely on IPTG supplementation. This indicates that replication cannot be initiated from an alternative RepN-independent origin. These results collectively argue against the possibility of alternative origin activation in our system.

Cells with replication arrested by CRISPRi^box6–7^ were characterized using fluorescence microscopy in combination with proteomic approaches. We found that CRISPRi-mediated blockade at the DnaA boxes 6 and 7 resulted in DNA replication arrest (Fig. 2), while protein production continued (Fig. 5B and 6). Replication-arrested cells were also longer than the WT cells (Fig. 3A and B), which is a hallmark of the SOS response and is typically mediated by the division inhibitor YneA (27). However, deleting *yneA* did not influence the cell length. Our analysis of SOS response-associated proteins in the proteomic data and the P*_yneA_*-GFP reporter for the SOS response did not indicate the presence of an active SOS stress response (Fig. S4). Both approaches yielded similar results, strongly suggesting that the phenotype observed was not driven by YneA, which is part of the SOS response (Fig. 4A and B).

After inducing the CRISPRi^box6–7^ system, we observed a complete halt in replication initiation after 3 h (Fig. 2). At the same time, the number of CFU per milliliter remained similar to the initial value at time point 0 (Fig. 1). However, the biomass continued to increase and the apparent growth began to halt around 5 h after induction (Fig. S1A). These results are consistent with the appearance of cells with very low DAPI fluorescence and the apparently longer cells observed already at 3 and 5 h after CRISPRi^box6–7^ induction (Fig. 3). We hypothesized that cells continue to elongate and divide, but only to a certain extent, after inhibiting replication initiation (Fig. 3). This could be detected by an increase in OD, while the CFU per milliliter did not increase. In addition, anucleated cells similar to those observed in *E. coli* (37) were observed (Fig. 3A).

Our results are consistent with the observations made by inhibiting replication initiation with a ts mutant of DnaB (20), where, after shifting to non-permissive temperatures, cells increased their mass during ~3 mass doubling periods. Cells arrested by *dnaBts*-mediated replication were also metabolically active and retained intact membranes. Interestingly, cell shape was also affected in a similar way to what we observed in Fig. 3A. The authors did not observe a reduction in FtsZ levels, but rather the cells were unable to form Z-rings. In this sense, our results show that this longer cell phenotype after replication arrest is not due to inhibition by YneA (Fig. 4) and the expression of genes in the operon encoding the cell division protein FtsL does not appear to be affected (Fig. S6), suggesting the existence of a secondary, different mechanism that partially stops cell division in an SOS response-independent manner. A few examples of SOS-independent inhibition of cell division have been reported in several microorganisms (38–40). However, no homolog of those systems has been reported in *B. subtilis*.

Proteomic analysis of non-replicating cells revealed that the reduction in the abundance of translation-related proteins (including rRNA-binding, ribosomal proteins, tRNA-binding and nucleoproteins) is more attenuated after 5 and 6 h post CRISPRi^box6–7^ induction, compared with the WT (Fig. 4A). Previous studies in several bacteria have shown that protein synthesis continues during growth arrest and suggested that the maintenance of a functional translation machinery may be required for their viability (31, 41–43). Moreover, in *E. coli*, several studies have demonstrated increased production of proteins and metabolites during replication arrest (32–34). Our study expands these observations by confirming that non-replicating cells can maintain translation (Fig. 5B and 6). It is intriguing that upon halting replication initiation, there was no generalized arrest of translation by downregulation of ribosome production or by the action of the (p)ppGpp alarmones. Interestingly, under normal growth conditions, when cells transition into stationary phase, the synthesis of the (p)ppGpp alarmone increases, inhibiting translation but at the same time replication (14). This second messenger can thus link a decrease in replication elongation to the downregulation of translation. However, the specific and artificial interference by CRISPRi^box6–7^ in the preceding step of replication initiation suggests that, under these conditions, there is no apparent coordination between the decrease in new replication rounds and the reduction of protein production.

Interestingly, we observed that GFP can be produced at similar levels in the non-replicating and WT strains; however, this protein amount is diluted in the larger cell volume of the replication-arrested cell. This is an interesting observation, and we hypothesize that replication-arrested cells possess only a single copy of the chromosome, in which transcription occurs at a similar rate as in the WT; these mRNAs are probably translated with similar efficiency in both strains, producing the same amount of total GFP. However, this amount diffuses in a cytoplasm of increased size, displaying an apparent reduced expression when the MFI of GFP is measured. This dilution effect due to increased cell size is corrected when measuring the total amount of GFP intensity per cell.

Non-replicating phenotypes with active translation hold considerable promise for industrial applications. Indeed, in the absence of replication, the utilization of carbon sources could be redirected from biomass production to the translation machinery. To make a *B. subtilis* system compatible with these potential industrial applications, it would be essential to design strategies that eliminate the necessity of adding xylose to induce dCas9 expression and that alleviate the constraints associated with cell growth. The expression of dCas9 can be controlled using self-inducing promoters (44–46), avoiding the reliance on chemical inducers, which may prove uneconomical on an industrial scale. With regard to the second challenge, preservation of cell shape and membrane homeostasis is crucial to prolong bacterial lifespan and avoid anticipated cell lysis in industrial contexts (26). Potential targets that inhibit cell replication and growth while enabling sustained protein production could be selected to meet this challenge in industrial applications. Similar approaches have been undertaken in *E. coli* (33).

Our study indicates that inhibiting DNA replication very specifically at an early time point by CRISPRi^box6–7^-mediated replication arrest does not immediately affect cell elongation and division, resulting in elongated cells that may lack genetic material. The translation is also not significantly reduced upon direct inhibition of DNA replication, suggesting that various signals at different levels trigger the coordinated control of cell size, replication initiation, and translation observed during post-exponential growth or stress conditions. Understanding this phenomenon could shed light on the natural phenotypes of non-growth and non-replication, and help in the development of antibiotics that take these non-growing states into account, or the improvement of microbial cell factories for industry.

## MATERIALS AND METHODS

### Strains and culture conditions

Bacterial strains and vectors used in this study are listed in Tables S1 and S2. *B. subtilis* and its derivative strains were routinely cultured in Luria-Bertani (LB Broth Miller, Becton Dickinson) medium containing 10 g/L tryptone, 5 g/L yeast extract, and 10 g/L NaCl (and 15 g/L agar for solid medium) with constant shaking of 180–200 rpm at 37°C. In addition, LB was supplemented with glucose (1%) or xylose (1%) when indicated. *E. coli* strains were grown at 37°C with constant shaking at 180 rpm in LB or LB agar supplemented with the appropriate antibiotics for selection on plates.

*B. subtilis* was grown at 37°C on LB agar plates streaked from bacterial glycerol stocks stored at −80°C. Subsequently, cultures were grown from single colonies. When needed, antibiotics were added to the media at the following final concentrations: 10 µg/mL kanamycin (Corning) and 1 µg/mL erythromycin (Sigma-Aldrich); 15 µg/mL lincomycin (Corning) and 150 µg/mL spectinomycin (Sigma-Aldrich). Xylose was added to a final concentration of 1% (wt/wt) to induce the conditional promoter (P*_xylA_*). Glucose was used at a final concentration of 1% (wt/vol) and was used to repress the conditional inducible xylose-inducible promoter (P*_xylA_*).

### Serial dilution plating viability assay

Overnight cultures were diluted to an OD_600_ of 0.01 in 20 mL LB with 1% glucose in 100 mL flasks and further incubated at 37°C, 180 rpm, until an OD_600_ of 0.25 (± 0.025). To ensure that cells were in early exponential phase, cultures were back-diluted 1:10 in a total volume of 120 mL LB and 1% glucose in 1 L flasks, incubated at 37°C, 180 rpm, until an OD_600_ of 0.1 (± 0.025). Cells were pelleted at 4,000 × *g* at room temperature for 5 min and resuspended in LB supplemented with either 1% glucose or 1% xylose. Cells were incubated at 37°C, 180 rpm, and 100 µL samples were taken at 0, 1, 3, and 20 h and then 10-fold serially diluted in LB. Five microliters of each dilution was spotted onto LB agar plates containing 1% (wt/vol) glucose to inhibit additional expression of dCas9 and incubated overnight at 37°C. The number of CFU per milliliter was monitored. Please consider that time point 0 for this and all the other experiments is taken immediately after the addition of xylose.

### Determination of origin‐to‐terminus ratio by qPCR

Cells in early logarithmic phase (OD_600_, 0.1 ± 0.025) were pelleted at 4,000 × *g* at room temperature for 5 min, split in two and resuspended either in LB supplemented with 1% glucose or 1% xylose, and incubated at 37°C, 180 rpm. Fifteen-milliliter samples were taken at different time points (0, 3, and 20 h), spun down at 11,000 × *g*, 4°C for 5 min, followed by a genomic DNA extraction with the Nucleospin DNA extraction kit (Macherey-Nagel) according to the manufacturer’s instructions.

The primer pair targeting the origin region was OLEC11491 and OLEC11492, and the primer pair targeting the terminus was OLEC11493 and OLEC11494. qPCR reactions of 20 µL contained 2.5 ng of DNA, 200 nM of each primer, and 10 µL of 2× Power SYBR Green PCR-Master-Mix (Applied Biosciences), and amplifications were performed using a QuantStudio 5 Real-Time PCR system (Applied Biosciences) according to the following protocol: 95°C for 3 min, followed by 40 cycles of 95°C for 30 s, 60°C for 30 s, and 72°C for 30 s. *ori-ter* ratios were analyzed using the 2∆∆Ct method (47). A fixed sample of the WT strain grown in late stationary phase, where the population would be expected to have an *ori-ter* ratio corresponding to 1, was used for normalization in each cycle.

### Replisome localization by GFP-DnaN

Cells in early logarithmic phase were pelleted at 4,000 × *g*, at room temperature for 5 min, and resuspended in LB supplemented with either 1% glucose or 1% xylose. Afterward, they were incubated at 37°C, 180 rpm, and 200 µL samples from time points 1, 2, 3, and 5 h were spun down at room temperature, 4,000 × *g* for 5 min. Cell pellets were washed twice with 1× phosphate-buffered saline (PBS) and resuspended in a final volume of 500 µL 1× PBS from which 1.5 µL was spotted onto 1.5% agarose pads and observed under the microscope using the GFP channel.

### DNA damage response assay

The promoter of *yneA* was fused to the *gfp* gene and integrated into the ectopic *amyE* locus (48). Strains in early logarithmic growth phase (OD_600_, 0.1) were treated with 1% glucose to repress or 1% xylose to induce expression of dCas9 for 3 h. Cells treated with 3 µg/mL mitomycin for 3 h were used as positive controls. One milliliter of each culture was washed twice (4,000 × *g*, room temperature for 5 min) and suspended in 1× PBS (NaCl 137 mM, KCl 27 mM, Na_2_HPO_4_ 10 mM, KH_2_PO_4_ 1.8 mM, pH 7.4); 2 µL was placed on 1.5% agarose pads buffered in Tris-acetate-EDTA (TAE).

### Fluorescence microscopy

Cell were grown to an OD_600_ of ∼0.4. One microliter of cells was spotted onto 1.5% agarose pads and imaged. Images were acquired with an Inverted Microscope (Leica DMi8, DFC9000 GT VSC-D6212 camera), and a 100× phase contrast objective (HC LP APO 100×/1.40 oil). Filter sets for GFP channel were used when indicated.

For cell staining, culture samples were taken 0, 3, and 5 h post-induction. Cells were harvested by centrifugation (4 min, 5,000 rpm at room temperature) and washed with 1 mL of 1× PBS and pelleted as above. The bacterial pellets were resuspended in 98 µL of 1× PBS and transferred to an amber tube. One microliter of 1 mg/mL DAPI and 1 µL of 0.1 mg/mL FM4-64 were added. After 10 min at 37°C, cells were harvested as described above and resuspended in 1× PBS + 1% (vol/vol) dimethyl sulfoxide (DMSO); 0.5 µL of this suspension was spotted on agar pads (1.5% low melting point agarose in 1× PBS + 1% DMSO). The images were taken with 50 ms exposure time for the phase contrast. To visualize the FM4-64 stain, we used 800 ms of exposure time (ex: 540 nm–580 nm, em: 592 nm–668 nm), and for the DAPI stain, 100 ms (ex: 327 nm–383 nm, em: 435 nm–485 nm). When required, GFP fluorescence signal was obtained with 100 ms of exposure time (ex: 450 nm–490 nm, em: 500 nm–550 nm). Microscopy images were processed using MicrobeJ (49) for cell segmentation and fluorescence intensity measurements. Cell volume was calculated by modeling cells as spherocylinders.

### Western blotting

Cell pellets after CRISPRi induction and control samples were resuspended in 100 mM HEPES pH 8 supplemented with 1 mM phenylmethylsulfonyl fluoride (PMSF) and lyzed by bead beating. Ten micrograms of cell lysates was resolved in Mini-PROTEAN TGX Stain-free AnyKD precast gels and transferred to 0.22 µm polyvinylidene fluoride (PVDF) membranes. Membranes were blocked with 5% skim milk in Tris-buffered saline buffer + 0.1% Tween-20 (TBS-T) and then incubated for 1 h with a 1:2,000 dilution of an anti-GFP horseradish peroxidase (HRP)-conjugated antibody (ab6663, Abcam) in 5% skim milk in TBS-T. Blots were developed using a chemiluminescent substrate (Thermo Scientific, 34577) and documented in a Fusion FX imaging system (Vilber).

### Identification and quantification of proteins by mass spectrometry

#### Sample collection

Strains were grown in LB supplemented with 1% glucose. Once they reached early logarithmic phase, cells were pelleted at 4,000 × *g*, room temperature for 5 min, and resuspended in LB supplemented with 1% xylose. Cells were incubated at 37°C, 180 rpm, and 20 mL samples were taken at different time points per treatment (0, 3, 4, 5, and 6 h) followed by centrifugation at 11,000 × *g*, 4°C for 5 min. Pellets were resuspended in 20 mL of ice-cold 1× PBS.

Cells were lyzed with 500 µL 2× lysis buffer [final concentration: 2% SDS, 20 mM Tris(2-carboxyethyl)phosphine (TCEP), 80 mM chloroacetamide (Sigma-Aldrich), 100 mM HEPES pH 8 (VWR), 0.5× protease inhibitors (Roche) in tubes containing 25 mg 0.1 mm silica beads (BeadBeater Glass beads), 0.1 mm (Roth)] and were disrupted using a FastPrep-24 5G Homogenizer (MP Biomedicals). Samples were spun down at 11,000 × *g*, 10 min, to separate the soluble from the insoluble fraction. The supernatant was heated at 95°C for 5 min and then cooled down to room temperature. The protein concentration was determined using the Pierce BCA Protein Assay Kit (ThermoFisher Scientific), followed by nucleic acid digestion with 0.5 units of Benzonase per 1 µg of protein (Sigma-Aldrich) for 30 min at 37°C. Samples were kept at −20°C for further analyses.

#### Sample preparation

Equal protein amounts of all samples were subjected to SP3 sample preparation (50) on an Agilent BRAVO liquid handling robot. Ten micrograms of a 1:1 mixture of hydrophilic and hydrophobic carboxyl-coated paramagnetic beads (SeraMag, #24152105050250 and #44152105050250, GE Healthcare) was added for each microgram of protein. Protein binding was induced by addition of acetonitrile to a final concentration of 50% (vol/vol). Samples were incubated for 10 min at room temperature. The tubes were placed on a magnetic rack, and beads were allowed to settle for 3 min. The supernatant was discarded, and beads were rinsed three times with 200 µL of 80% ethanol without removing the tubes from the rack. Beads were resuspended in digestion buffer containing 50 mM triethylammonium bicarbonate and both trypsin (Serva) and Lys-C (Wako) in a 1:50 enzyme to protein ratio. Protein digestion was carried out for 14 h at 37°C in a PCR cycler. Afterward, the supernatant was recovered and dried down in a vacuum concentrator.

#### Peptide labeling and fractionation

Tandem mass tag (TMT) 11plex (Pierce, #A37725) was used for peptide multiplexing and quantification. Briefly, equal amounts of peptides were resuspended in 50 mM HEPES, pH 8.5. Additionally, 10% from each sample was pooled to create a common sample as internal standard. TMT reagents were allowed to equilibrate at room temperature for 30 min and were dissolved in anhydrous acetonitrile to a final concentration of 59 mM. TMT was added to each sample to a final concentration of 11.8 mM and tubes were incubated at 25°C for 60 min with mixing at 500 rpm on a ThermoMixer. Labeling was quenched by addition of hydroxylamine to a final concentration of 0.4%. Samples were mixed, desalted using solid phase extraction (Sep-Pak 1 cc/50 mg, Waters), dried down in a vacuum concentrator, and resuspended in 20 µL 2% acetonitrile. Basic reversed phase fractionation was performed on a quaternary Agilent 1290 Infinity II UPLC System equipped with a Kinetex Evo-C18 column (150 × 2.1 mm, 2.6 µm, 100 Å, Phenomenex) that was operated at 40°C. Solvent A consisted of high pressure liquid chromatography (HPLC) grade water, solvent B consisted of 100% acetonitrile, and solvent C consisted of 25 mM ammonium bicarbonate in water. Fractionation was carried out at a constant flow rate of 100 µL/min using a linear gradient from 2% to 25% acetonitrile within 50 min, followed by column washing and equilibration. Over the whole gradient, solvent C was kept constant at 10%. In total, 32 fractions were collected in conical 96-well plates. The organic solvent was removed in a vacuum concentrator for 30 min and fractions were concatenated into eight final samples. Peptides were acidified with formic acid, desalted using OASIS HLB 96-well cartridges (Waters, #186001828BA), dried down, and resuspended in 2% acetonitrile, 0.1% trifluoroacetic acid (TFA) prior mass spectrometry (MS) analysis.

#### Mass spectrometry

All samples were analyzed on a Orbitrap Exploris (Thermo Scientific) that was coupled to a 3000 RSLCnano UPLC (Thermo Scientific). Samples were loaded on a PepMap trap cartridge (300 µm I.D. × 5 mm, C18, Thermo) with 2% acetonitrile, 0.1% TFA at a flow rate of 20 µL/min. Peptides were separated over a 50 cm analytical column (Picofrit, 360 µm O.D., 75 µm I.D., 10 µm tip opening, non-coated, New Objective) that was packed in-house with Poroshell 120 EC-C18, 2.7 µm (Agilent). Solvent A consists of 0.1% formic acid in water. Elution was carried out at a constant flow rate of 250 nL/min using a 180-min method: 8%–33% solvent B (0.1% formic acid in 80% acetonitrile) within 120 min, 33%–48% solvent B within 25 min, 48%–98% buffer B within 1 min, followed by column washing and equilibration. Data acquisition on the Orbitrap Exploris was carried out using a data-dependent method in positive ion mode. MS survey scans were acquired from 375 to 1,500 *m*/*z* in profile mode at a resolution of 120,000. Automatic gain control (AGC) target was set to 100% at a maximum injection time of 50 ms. The 10 most abundant peptides were isolated within a 0.4 *m*/*z* window and subjected to higher-energy collisional dissociation (HCD) fragmentation at a normalized collision energy of 36%. The MS2 AGC target was set to 200%, allowing a maximum injection time of 54 ms. Product ions were detected in the Orbitrap at a resolution of 30,000. TurboTMT acquisition was enabled. Precursors were dynamically excluded for 45 s.

#### Data analysis

Data analysis was performed as described in Schäfer and collaborators (51). A two-tailed *t*-test and *P*-value correction were performed using Perseus to identify differentially expressed proteins (|log_2_ fold change| ≥ 1; *P*-value ≤0.05). Gene set enrichment analysis was performed using the fgsea package in R. Gene sets were obtained from SubtiWiki (52). Proteins forming part of the ribosome or involved in cell length, shape, or cell division used for profile plots and heat maps were retrieved from SubtiWiki (52). The log_2_ protein intensities were scaled to standard deviation units (*z*-scores) using R for profile plots.

## Data Availability

The mass spectrometry proteomics data have been deposited to the ProteomeXchange Consortium via the PRIDE (53) partner repository with the data set identifier PXD036876.

## SUPPLEMENTAL MATERIAL

Figure S1 msystems.00221-24-s0001.docx Optical density and morphology of replication arrested cells.

Figure S2 msystems.00221-24-s0002.docx Proteomic response of *B. subtilis* to replication arrest.

Figure S3 msystems.00221-24-s0003.docx Protein abundance of the *dnaA-dnaN* operon.

Figure S4 msystems.00221-24-s0004.docx No evidence of SOS response under replication arrest.

Figure S5 msystems.00221-24-s0005.docx No evidence of Sigma B-mediated stress under replication arrest.

Figure S6 msystems.00221-24-s0006.docx Protein abundance of genes regulated by DnaA.

Figure S7 msystems.00221-24-s0007.docx Abundance of proteins involved in cell shape, cell division, DNA segregation, and peptidoglycan biosynthesis.

Figure S8 msystems.00221-24-s0008.docx Gene set enrichment analysis of differentially expressed genes in CRISPRi box6-7 and WT strains over time.

Tables S1-S3 msystems.00221-24-s0009.docx Strains, plasmids, and oligonucleotides.

ASM does not own the copyrights to Supplemental Material that may be linked to, or accessed through, an article. The authors have granted ASM a non-exclusive, world-wide license to publish the Supplemental Material files. Please contact the corresponding author directly for reuse.
