# Supplementary material for: Bacillus subtilis remains translationally active after CRISPRi-mediated replication initiation arrest
Source: mSystems. 2024 Mar 28;9(4):e00221-24. doi: 10.1128/msystems.00221-24 (PMC11019786; doi:10.1128/msystems.00221-24)
Supplement: Figure S1 — Optical density and morphology of replication arrested cells. [file msystems.00221-24-s0001.docx]

**
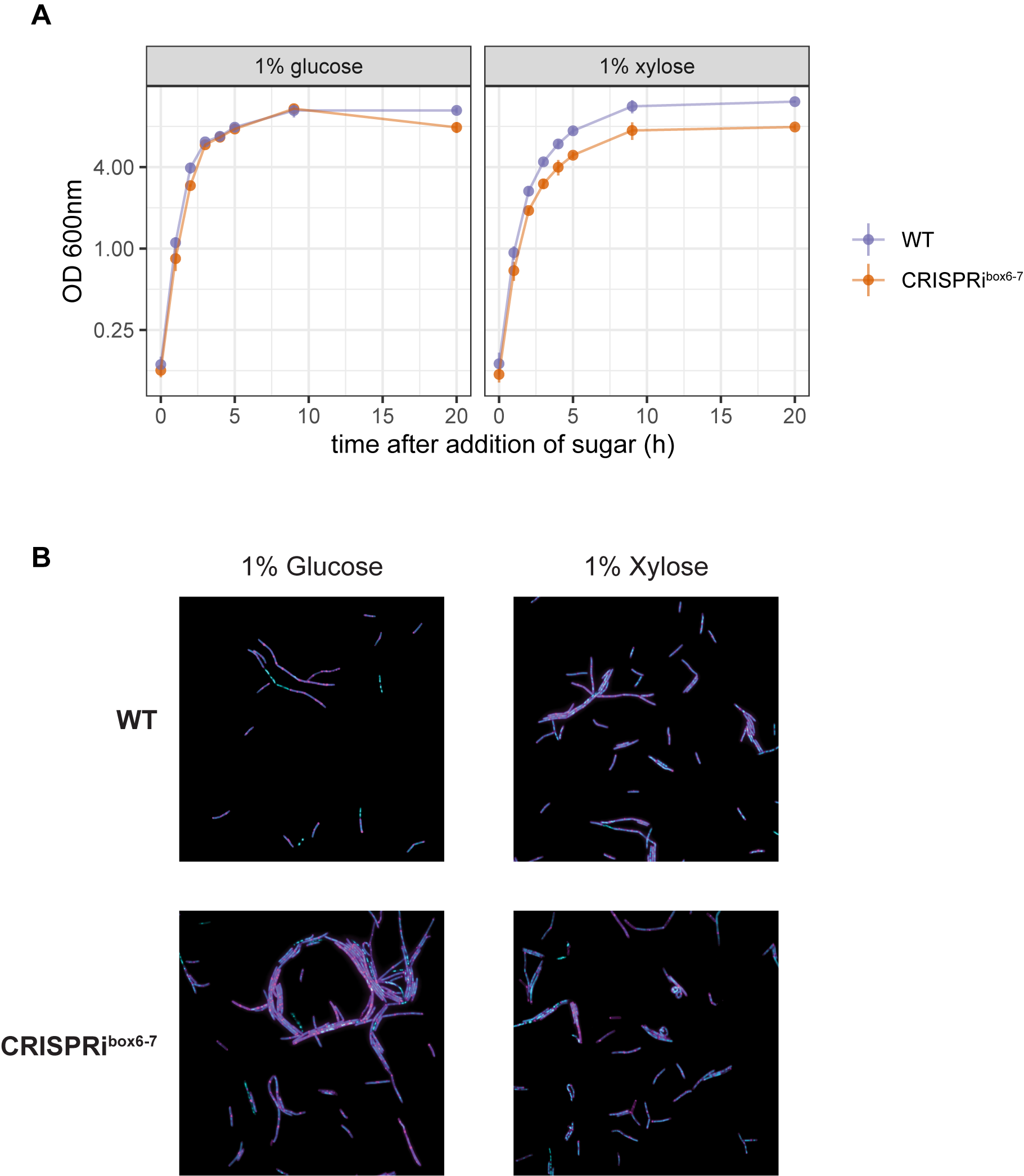
**

**Figure S1 Optical density and morphology of replication arrested cells. A)** Optical density of the cells under repression (glucose) or induction (xylose) conditions. Data shown are the mean of three independent biological replicates; the error bars represent standard deviations from the mean. **B)** Cell morphology of WT and CRISPRi strains at timepoint 0 stained with DAPI (cyan) and FM4-64 (magenta) and resuspended in LB with 1% glucose (repression) or 1% xylose (induction). Representative picture of three independent biological replicates.
