# Supplementary material for: Bacillus subtilis remains translationally active after CRISPRi-mediated replication initiation arrest
Source: mSystems. 2024 Mar 28;9(4):e00221-24. doi: 10.1128/msystems.00221-24 (PMC11019786; doi:10.1128/msystems.00221-24)
Supplement: Figure S2 — Proteomic response of B. subtilis to replication arrest. [file msystems.00221-24-s0002.docx]

**
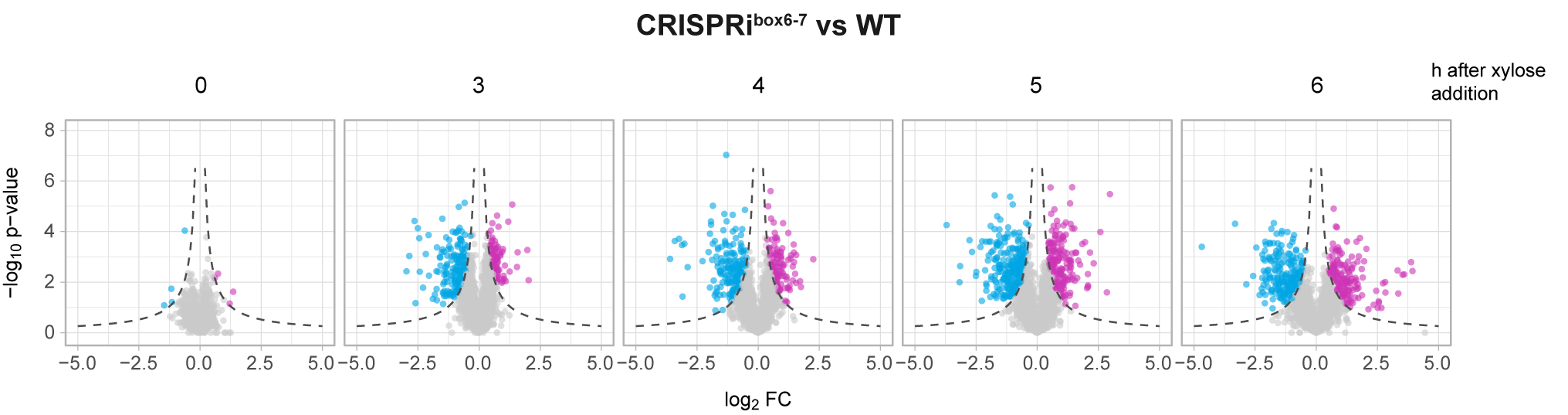
**

**Figure S2 Proteomic response of *B. subtilis* to replication arrest.** Volcano plot of the WT strain versus the CRISPRi^box6-7^ strain, showing the statistical significance (*p*-value) versus magnitude of change (fold change). Each protein is represented as a dot. Proteins whose differential expression is statistically significant are highlighted.
