# Supplementary material for: Bacillus subtilis remains translationally active after CRISPRi-mediated replication initiation arrest
Source: mSystems. 2024 Mar 28;9(4):e00221-24. doi: 10.1128/msystems.00221-24 (PMC11019786; doi:10.1128/msystems.00221-24)
Supplement: Figure S3 — Protein abundance of the dnaA-dnaN operon. [file msystems.00221-24-s0003.docx]

**
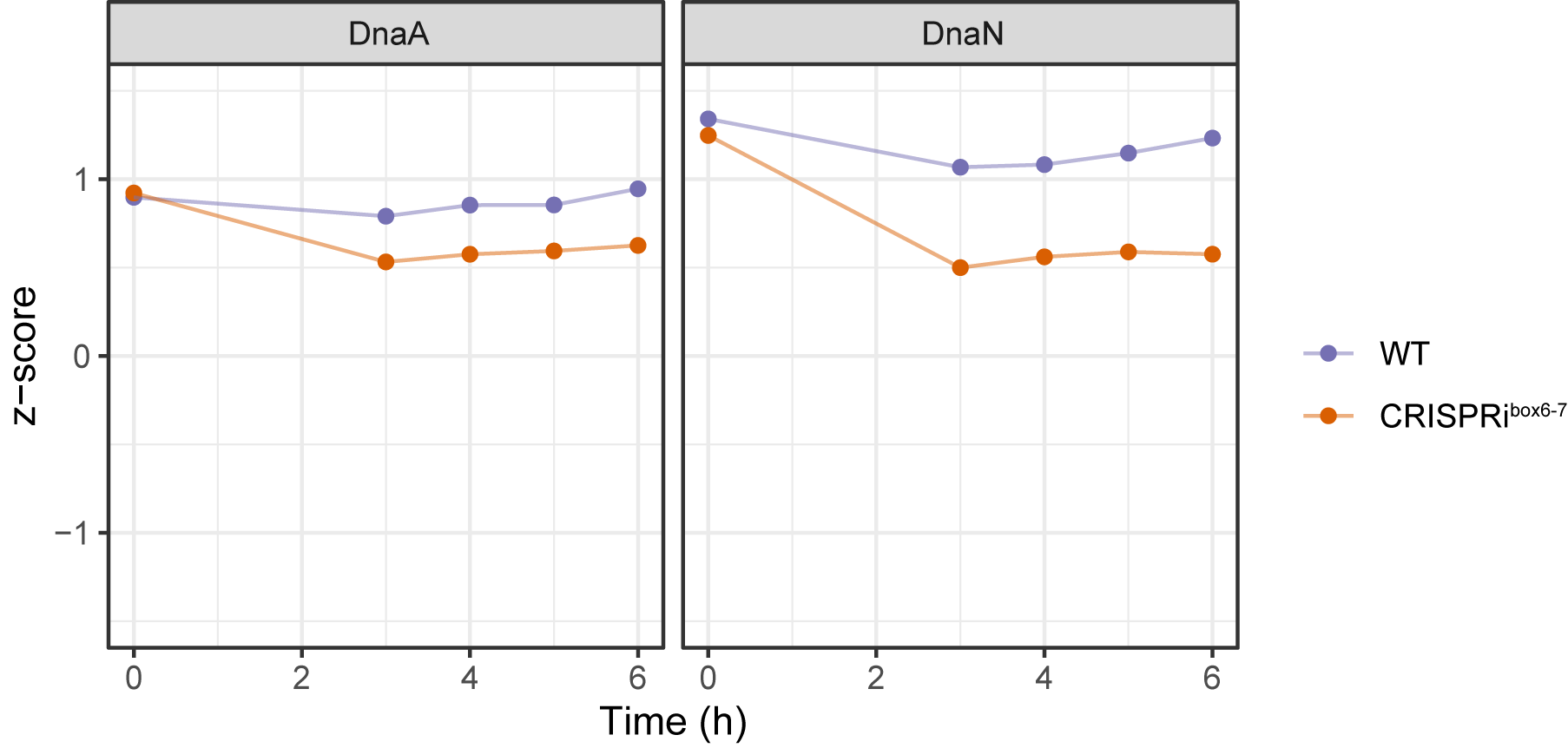
**

**Figure S3 Protein abundance of the *dnaA-dnaN* operon.** Profile plots of z-score-normalized protein intensities of DnaA and DnaN. Data obtained from the MS analysis.
