# Supplementary material for: Bacillus subtilis remains translationally active after CRISPRi-mediated replication initiation arrest
Source: mSystems. 2024 Mar 28;9(4):e00221-24. doi: 10.1128/msystems.00221-24 (PMC11019786; doi:10.1128/msystems.00221-24)
Supplement: Figure S4 — No evidence of SOS response under replication arrest. [file msystems.00221-24-s0004.docx]

***
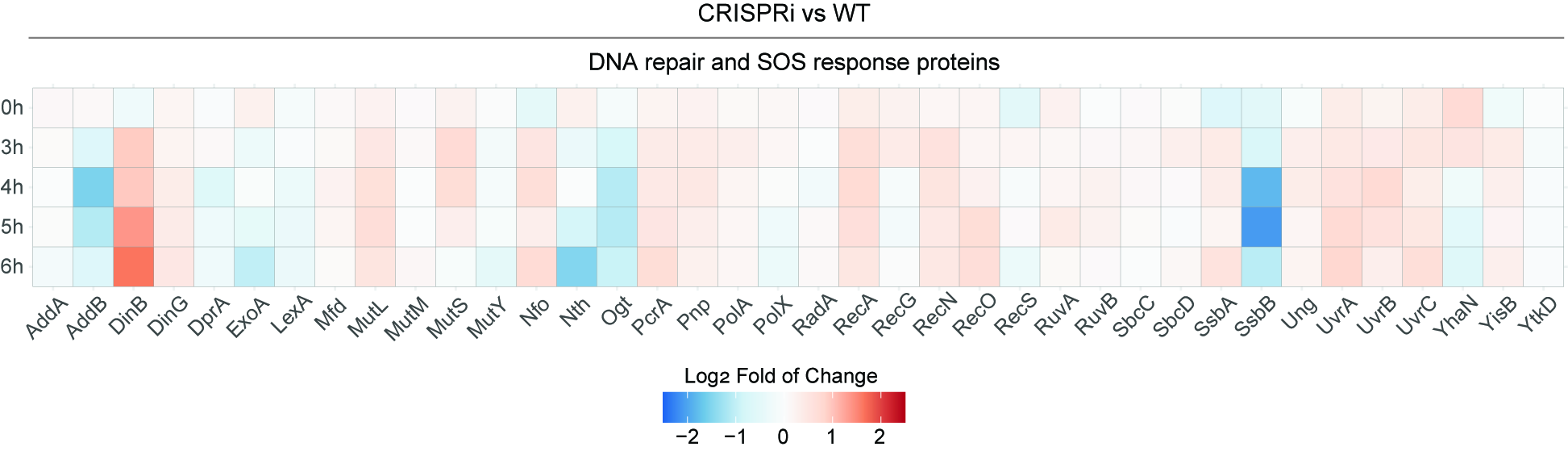
***

**Figure S4 No evidence of SOS response under replication arrest.** Heatmap based on fold change values of selected proteins associated with DNA repair and SOS response. Protein annotation was retrieved from SubtiWiki. Selected data represent the mean of three independent biological replicates.
