# Supplementary material for: Bacillus subtilis remains translationally active after CRISPRi-mediated replication initiation arrest
Source: mSystems. 2024 Mar 28;9(4):e00221-24. doi: 10.1128/msystems.00221-24 (PMC11019786; doi:10.1128/msystems.00221-24)
Supplement: Figure S5 — No evidence of Sigma B-mediated stress under replication arrest. [file msystems.00221-24-s0005.docx]

**
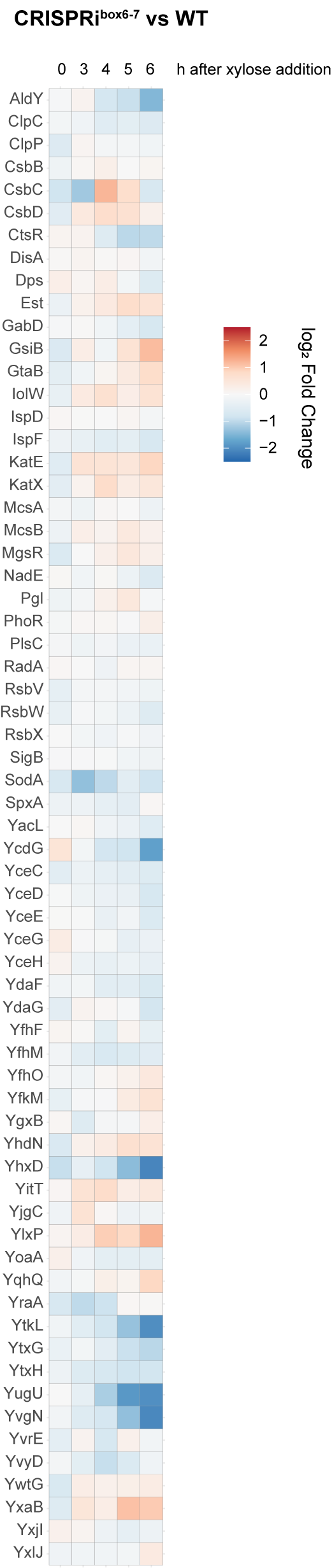
**

**Figure S5 No evidence of Sigma B-mediated stress under replication arrest.** Heatmap based on fold change values of proteins related to Sigma B regulon. All proteins annotations are retrieved from SubtiWiki.
