# Supplementary material for: Bacillus subtilis remains translationally active after CRISPRi-mediated replication initiation arrest
Source: mSystems. 2024 Mar 28;9(4):e00221-24. doi: 10.1128/msystems.00221-24 (PMC11019786; doi:10.1128/msystems.00221-24)
Supplement: Figure S6 — Protein abundance of genes regulated by DnaA. [file msystems.00221-24-s0006.docx]

**
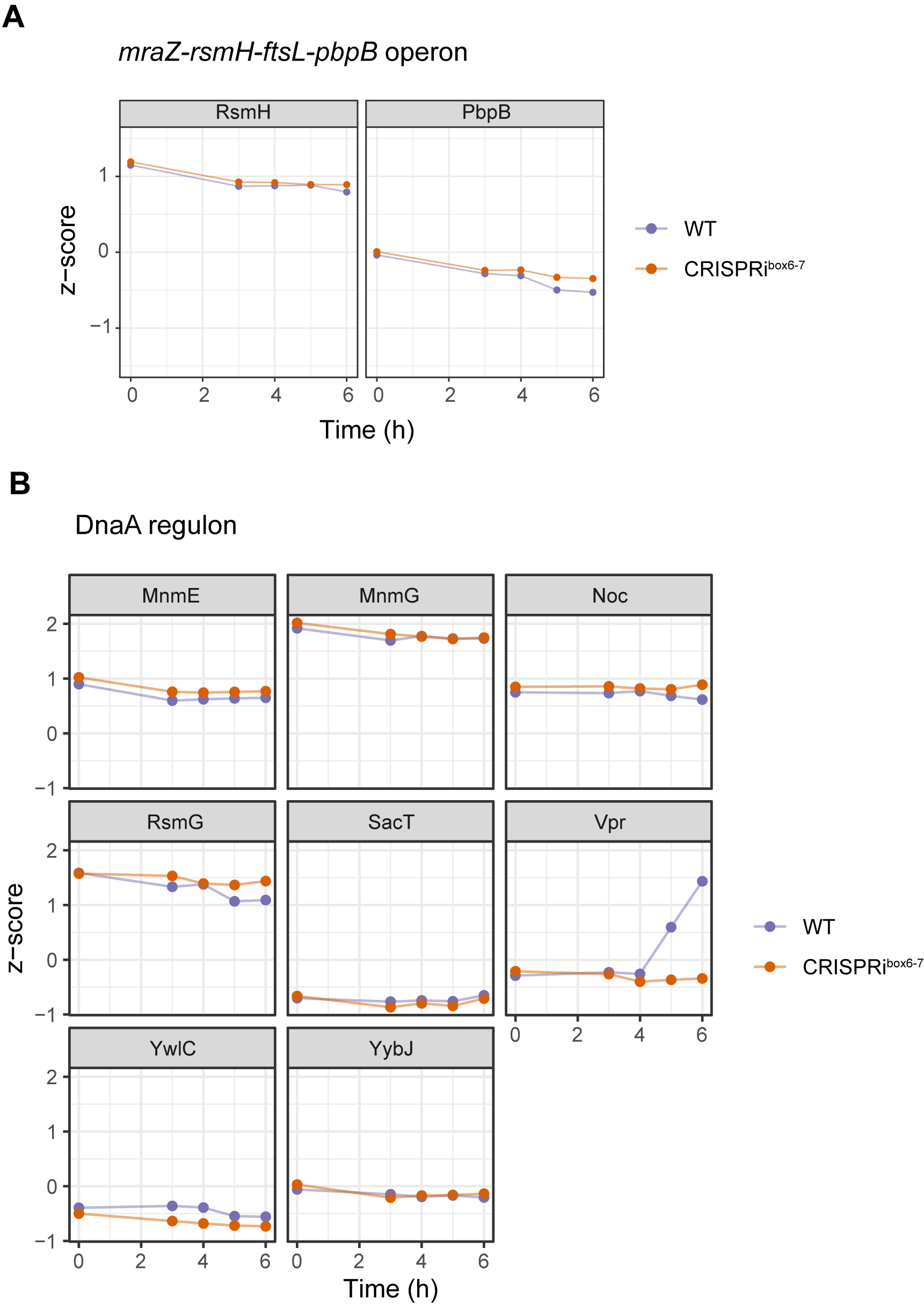
**

**Figure S6 Protein abundance of genes regulated by DnaA. A)** Profile plots of the two other proteins in the *ftsL* operon, PbpB and RsmH. **B)** Profile plots of other proteins whose coding genes are regulated by DnaA. The DnaA regulon members were obtained from SubtiWiki. Data obtained from the MS analysis.
