# Supplementary material for: Bacillus subtilis remains translationally active after CRISPRi-mediated replication initiation arrest
Source: mSystems. 2024 Mar 28;9(4):e00221-24. doi: 10.1128/msystems.00221-24 (PMC11019786; doi:10.1128/msystems.00221-24)
Supplement: Figure S7 — Abundance of proteins involved in cell shape, cell division, DNA segregation, and peptidoglycan biosynthesis. [file msystems.00221-24-s0007.docx]

**
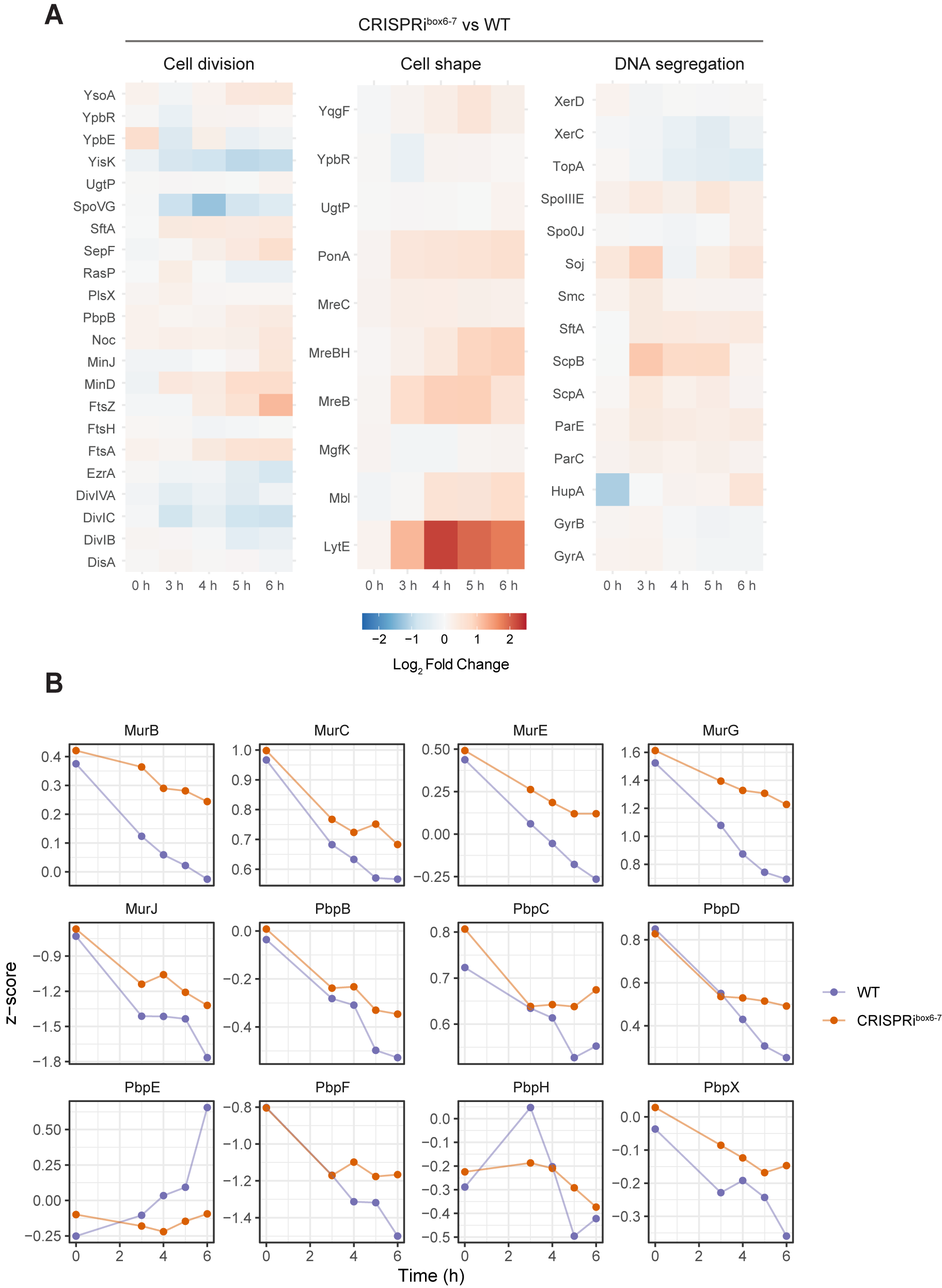
**

**Figure S7 Abundance of proteins involved in cell shape, cell division, DNA segregation and peptidoglycan biosynthesis.** **A)** Heatmap based on log_2_ fold change values of proteins related to cell division, cell shape and DNA segregation. All proteins annotations are retrieved from SubtiWiki. B) Profile plots of z-scored protein abundances of proteins involved in peptidoglycan precursor biosynthesis and penicillin-binding proteins.
