# Supplementary material for: Bacillus subtilis remains translationally active after CRISPRi-mediated replication initiation arrest
Source: mSystems. 2024 Mar 28;9(4):e00221-24. doi: 10.1128/msystems.00221-24 (PMC11019786; doi:10.1128/msystems.00221-24)
Supplement: Figure S8 — Gene set enrichment analysis of differentially expressed genes in CRISPRi box6-7 and WT strains over time. [file msystems.00221-24-s0008.docx]

**
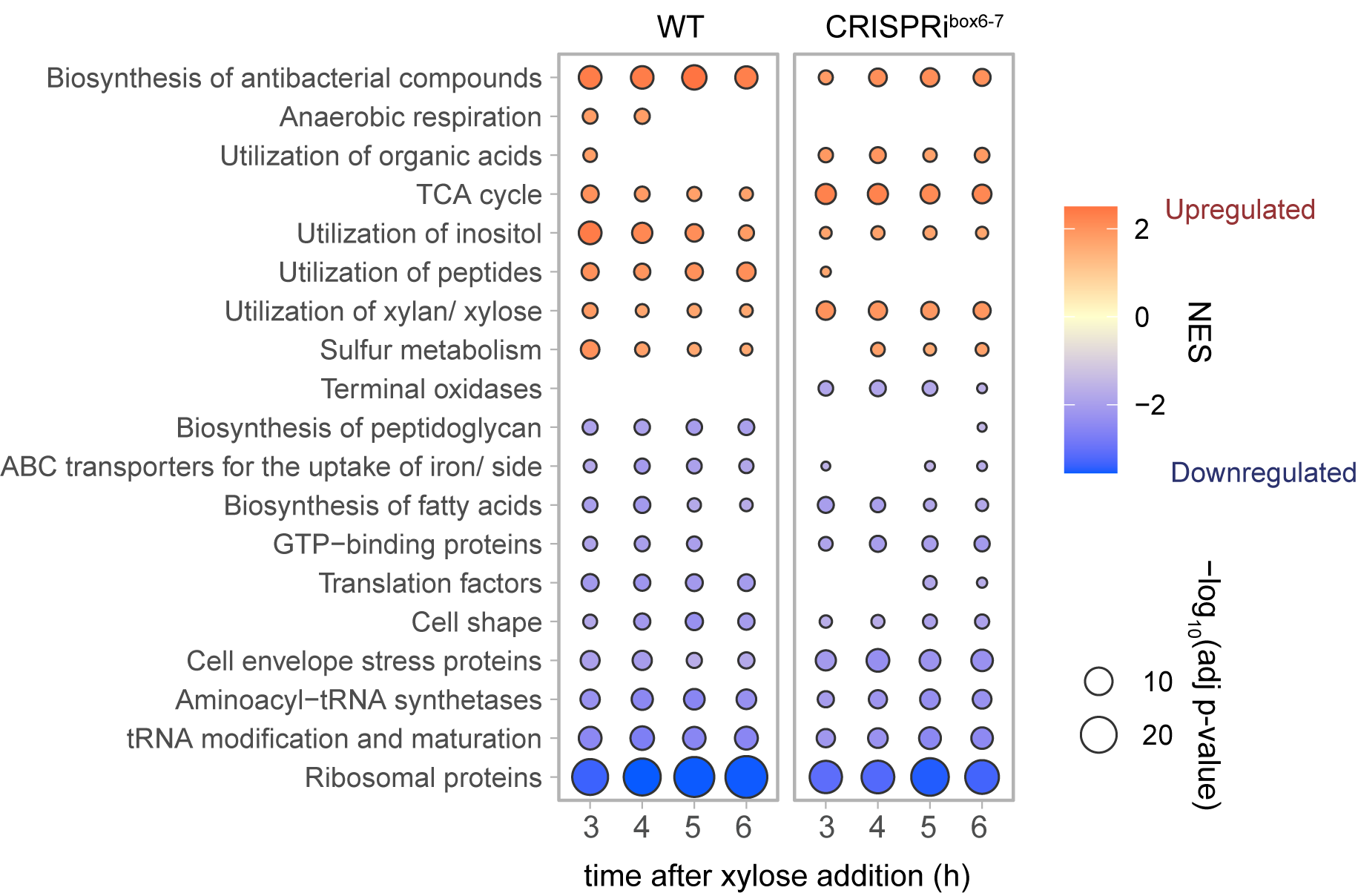
**

**Figure S8 Gene set enrichment analysis of differentially expressed genes in CRISPRi^box6-7^ and WT strains over time.** Each time point was compared to the time point 0. Gene categories were obtained from Subtiwiki. The color of the dots shows the normalized enrichment score (NES) values for each process, and the size of the dots represents the -log_10_ of the adjusted *p*-value.
