# Supplementary material for: Bacillus subtilis remains translationally active after CRISPRi-mediated replication initiation arrest
Source: mSystems. 2024 Mar 28;9(4):e00221-24. doi: 10.1128/msystems.00221-24 (PMC11019786; doi:10.1128/msystems.00221-24)
Supplement: Tables S1-S3 — Strains, plasmids, and oligonucleotides. [file msystems.00221-24-s0009.docx]

**Table S1. Bacterial strains used in this study**

| Strain | Relevant characteristics | Source |
| --- | --- | --- |
| *Escherichia coli* strains – Hosts for cloning | | |
| TOP10 | K12 strain, F^–^ *mcrA* Δ*(mrr-hsdRMS-mcrBC)* φ80d*lacZ*Δ*M15* Δ*lacX74 recA1 araD139* Δ*(ara-leu)7697 galU galK* λ^–^ *rpsL endA1 nupG*; SmR | Invitrogen |
| NEB 5-alpha | K12 strain, *fhuA2*::IS2 Δ*(mmuP-mhpD)169* Δ*phoA8 glnX44* φ80d[Δ*lacZ58(M15)*] *rfbD1 gyrA96 luxS11 recA1 endA1 rph*^WT^ *thiE1 hsdR17* | NEB, Anton & Raleigh, 2016 (1) |
| *Bacillus subtilis* strains | | |
| EC3017  (WT) | PY79  wildtype, *trpC2* prototroph | Youngman et al, 1984 (2) |
| EC3137  (WT-dCas9) | EC3017 *lacA*::P*_xyl_*-*dcas9* (Erm^R^) | This work |
| EC3146  (CRISPRi^box1-2^) | EC3137 *amyE*::P*_veg_*-sgRNA^box1-2^  (Cm^R^, Erm^R^) | This work |
| EC3147  (CRISPRi^box6-7^) | EC3137 *amyE*::P*_veg_*-sgRNA^box6-7^ (Cm^R^, Erm^R^) | This work |
| EC3149  (CRISPRi^box3-4^) | EC3137 *amyE*::P*_veg_*-sgRNA^box3-4^ (Cm^R^, Erm^R^) | This work |
| EC3686  (CRISPRi^nt^) | EC3137 *lacA*::P*_xyl_*-dCas9, *amyE*::P_veg_-non-targeting sgRNA (Cm^R^, Erm^R^) | This work |
| EC3237 | EC3017 P*_dnaAN_-dnaA-gfp-dnaN (*Cm^R^) | This work  Adapted from (3) |
| EC3259 | EC3017 *amyE*::P*_veg_*-sgRNA^box6-7^, *lacA*::P*_xyl_*-*dcas9*, *gfp-dnaN* (Spc^R^, Erm^R^, Cm^R^) | This work |
| EC3266 | EC3017 *amyE*::P*_yneA_*-*gfp-yneA* (Cm^R^) | This work  Adapted from (4) |
| EC3270 | EC3017 *amyE*:: P*_yneA_*-*gfp-yneA,* *thrC*::P*_veg_*-sgRNA^box6-7^ (Cm^R^, Spc^R^) | This work |
| EC3272 | EC3270 *lacA*::P*_xyl_*-*dcas9* (Erm^R^) | This work |
| EC3673 | EC3017 *amyE*::P*_veg_*-sgRNA^box6-7^, *thrC*::P*_hyperspank_*-*gfpmut2*, *lacA*::P*_xyl_*-*dcas9* (Erm^R^, Spc^R^, Cm^R^) | This work |
| EC3698 | EC3673 Δ*yneA*::*lox71*-KanR-*lox66* (Erm^R^, Spc^R^, Cm^R^) | This work |
| EC3700 | EC3017 *thrC*::P*_hyperspank_*-*gfpmut2* (Spc^R^) | This work |
| EC3704 | EC3700 Δ*yneA*::*lox71*-KanR-*lox66* (Kan^R^, Spc^R^) | This work |

**Abbreviations:** Antibiotic resistances are represented as: Amp^R^ – ampicillin, Cm^R^ – chloramphenicol, Erm^R^ – erythromycin, Kan^R^ – kanamycin, Spc^R^ – spectinomycin.

**Table S2. Plasmids used in this study**

| Plasmid | Relevant characteristics^b^ | Source |
| --- | --- | --- |
| pEC2728 (pJMP1) | *B. subtilis* *lacA*/*ganA* integrative vector  *pAX01*Ω*P_xyl_-dcas9* (Erm^R^) | Addgene #79873 (5) |
| pEC2728 (pJMP2) | *B. subtilis* *amyE* integrative vector  *pDG1662ΩP_veg_-sgRNA_RR1* (Cm^R^) | Addgene #79874 (5) |
| pEC2730 (pJMP3) | *B. subtilis* *thrC* integrative vector  *pDG1731* ΩP*_veg_-sgRNA_RR1* (Spc^R^) | Addgene #79875 (5) |
| pEC2741 | *B. subtilis* *amyE* integrative vector  *pDG1662ΩP_veg_-sgRNA*^box1-2^ (Cm^R^) | This work |
| pEC2742 | *B. subtilis* *amyE* integrative vector  *pDG1662ΩP_veg_-sgRNA*^box6-7^ (Cm^R^) | This work |
| pEC2743 | *B. subtilis* *amyE* integrative vector  *pDG1662ΩP_veg_-sgRNA*^box3-4^ (Cm^R^) | This work |
| pEC2892 | *B. subtilis* *thrC* integrative vector  *pDG1731* ΩP*_veg_-sgRNA*^box6-7^ (Spc^R^) | This work |
| pEC2990 | pEC2730 *thrC*::P*_hyperspank_*-*gfpmut2* (Spc^R^) | This work |

**Table S3. Oligonucleotides used for marker frequency analysis**

| Oligo | Sequence 5′-3′ | F/R | Purpose/ Target |
| --- | --- | --- | --- |
| OLEC11491 | GATCAATCGGGGAAAGTGTG | F | qPCR *ori-ter* ratio |
| OLEC11492 | GTAGGGCCTGTGGATTTGTG | R | qPCR *ori-ter* ratio |
| OLEC11493 | TCCATATCCTCGCTCCTACG | F | qPCR *ori-ter* ratio |
| OLEC11494 | ATTCTGCTGATGTGCAATGG | R | qPCR *ori-ter* ratio |

F/R - Forward/Reverse. All oligonucleotides were ordered from Sigma-Aldrich.

**References**

1. Anton BP, Raleigh EA. 2016. Complete genome sequence of NEB 5-alpha, a derivative of *Escherichia coli* K-12 DH5α. Genome Announc 4:10.1128.

2. Youngman P, Perkins JB, Losick R. 1984. Construction of a cloning site near one end of Tn917 into which foreign DNA may be inserted without affecting transposition in *Bacillus subtilis* or expression of the transposon-borne *erm* gene. Plasmid 12:1–9.

3. Aakre CD, Phung TN, Huang D, Laub MT. 2013. A bacterial toxin inhibits DNA replication elongation through a direct interaction with the β sliding clamp. Mol Cell 52:617–628.

4. Gozzi K, Ching C, Paruthiyil S, Zhao Y, Godoy-Carter V, Chai Y. 2017. *Bacillus subtilis* utilizes the DNA damage response to manage multicellular development. NPJ Biofilms Microbiomes 3:8.

5. Peters JM, Colavin A, Shi H, Czarny TL, Larson MH, Wong S, Hawkins JS, Lu CHS, Koo B-M, Marta E, Shiver AL, Whitehead EH, Weissman JS, Brown ED, Qi LS, Huang KC, Gross CA. 2016. A comprehensive, CRISPR-based functional analysis of essential genes in Bacteria. Cell 165:1493–1506.
